# A toolbox for calculating quantitative image properties in aesthetics research

**DOI:** 10.3758/s13428-025-02632-3

**Published:** 2025-03-14

**Authors:** Christoph Redies, Ralf Bartho, Lisa Koßmann, Branka Spehar, Ronald Hübner, Johan Wagemans, Gregor U. Hayn-Leichsenring

**Affiliations:** 1 Experimental Aesthetics Group, Institute of Anatomy I, Universitätsklinikum Jena, Friedrich-Schiller-Universität Jena, Teichgraben 7, 07743 Jena, Germany; 2 Laboratory of Experimental Psychology, Department of Brain and Cognition, University of Leuven (KU Leuven), Leuven, Belgium; 3 School of Psychology, University of New South Wales, Sydney, Australia; 4 Department of Psychology, University of Konstanz, Konstanz, Germany

**Keywords:** Image features, Toolbox, Image analysis, Convolutional neural networks, Fourier analysis, Box counting, Fractality, Entropy, Self-similarity, Complexity, Symmetry, Color

## Abstract

Over the past two decades, researchers in the field of visual aesthetics have studied numerous quantitative (objective) image properties and how they relate to visual aesthetic appreciation. However, results are difficult to compare between research groups. One reason is that researchers use different sets of image properties in their studies. However, even if the same properties are used, the image pre-processing techniques may differ, and researchers often use their own customized scripts to calculate the image properties. To provide better accessibility and comparability of research results in visual experimental aesthetics, we developed an open-access and easy-to-use toolbox called *Aesthetics Toolbox*. The Toolbox allows users to calculate a well-defined set of quantitative image properties popular in contemporary research. The properties include image dimensions, lightness and color statistics, complexity, symmetry, balance, Fourier spectrum properties, fractal dimension, self-similarity, as well as entropy measures and CNN-based variances. Compatible with most devices, the Toolbox provides an intuitive click-and-drop web interface. In the Toolbox, we integrated the original scripts of four different research groups and translated them into Python 3. To ensure that results were consistent across analyses, we took care that results from the Python versions of the scripts were the same as those from the original scripts. The toolbox, detailed documentation, and a link to the cloud version are available via GitHub: https://github.com/RBartho/Aesthetics-Toolbox. In summary, we developed a toolbox that helps to standardize and simplify the calculation of quantitative image properties for visual aesthetics research.

## Introduction

Many contemporary models of aesthetic experience hypothesize that aesthetic judgments by human beholders are partially based on the perception of low- and mid-level visual features (Datta et al., 2006; Farzanfar & Walther, 2023; Gómez-Puerto et al., 2016; Graham & Redies, 2010; Ibarra et al., 2017; Li & Chen, 2009; Li & Zhang, 2020; Nakauchi et al., 2022; Palmer et al., 2013; Peng, 2022; Redies, 2015; Sidhu et al., 2018; Taylor et al., 2011; Zhang et al., 2021). Besides perceptual processing, cognition and emotions are considered to be crucial determinants of aesthetic experience (“aesthetic triad”; Chatterjee & Vartanian, 2014; Hekkert, 2006). In support of the role of perceptual processes, quantitative (objective) image properties were found to predict aesthetic judgments such as ratings of liking, interest, or beauty on different types of visual stimuli, including artworks (for reviews, see Brachmann & Redies, 2017a; Chamberlain, 2022; Leder et al., 2004; Redies, 2007; Spehar et al., 2015; Taylor et al., 2011).

In the present article, we describe a toolbox called the “Aesthetics Toolbox” that can compute a set of quantitative (objective) image properties studied in aesthetics research previously. The image properties are calculated based on the physical structure of digital images and are therefore independent of the beholder. An overview of all image properties of the Toolbox is given in Table 1. By contrast, subjective image properties are based on the impressions or feelings that images evoke in beholders (Chamberlain, 2022; Lyssenko et al., 2016). Subjective human responses to images can be measured, e.g., by psychological methods. When we refer to image properties in the present work, we mean quantitative (objective) image properties, unless stated otherwise. Note that in the field of computer vision, image properties are usually referred to as image features.

**Table 1:** Overview of quantitative image properties (QIPs) calculated by the Toolbox

Type of property	Name	Measured Property (algorithm used)	Reference(s)
Image dimensions	Image size	Sum of height and width of image (default method)	Datta et al. (2006)
	Aspect ratio	Ratio of width to height of image	Datta et al. (2006)
Contrast, lightness entropy, and complexity	RMS contrast	Standard deviation of the L* channel of the L*a*b* color space	Peli (1990)
	Lightness entropy	Shannon entropy of the L* channel of the L*a*b* color space	Kersten (1987), Mather (2018), Shannon (1948)
	Complexity (HOG)	Mean value over the combined luminance and color gradient images (HOG features)	Braun et al. (2013), Dalal and Triggs (2005)
	Edge density	Edge density of Gabor-filtered image	Mehrotra et al. (1992), Redies et al. (2017)
Color	Channel mean	Mean values for each channel of the RGB, L*a*b*, and HSV color spaces, respectively	Geller et al. (2022), Li and Chen (2009), Mallon et al. (2014), Sidhu et al. (2018)
	Channel SD	Standard deviations for each channel of the RGB, L*a*b*, and HSV color spaces, respectively	Geller et al. (2022), Sidhu et al. (2018)
	Color entropy	Shannon entropy of the H channel of the HSV color space	Geller et al. (2022), Shannon (1948)
Balance and Symmetry	Balance	Assessment of Preference for Balance (APB) score	Hübner and Fillinger (2016), Wilson and Chatterjee (2005)
	DCM	Deviation of the center-of-mass	Hübner and Fillinger (2016), McManus et al. (2011)
	Mirror symmetry	Mean of mirror symmetry measures around different axes	Hübner and Fillinger (2016), Wagemans (1995)
	Symmetry (CNN)	CNN-feature based symmetry	Brachmann and Redies (2016), Krizhevsky et al. (2012)
Scale invariance and self-similarity	Fourier spectrum Slope	Slope of log-log plots of the spatial frequency amplitude or power (Fourier) spectrum	Burton and Moorhead (1987), Isherwood et al. (2021), Mather (2014), Redies et al. (2007), Spehar et al. (2003)
	Fourier spectrum Sigma	Deviation of measured data points from regression line in log-log plots of the spatial frequency power (Fourier) spectrum	Redies et al. (2007)
	2D fractal dimension *D*	Two-dimensional fractal dimension* D* (binarized image; box-counting method)	Taylor et al. (1999), Viengkham and Spehar (2018)
	3D fractal dimension *D*	Three-dimensional fractal dimension *D* (grayscale image; box-counting method)	Liu et al. (2014), Mather (2018)
	Self-similarity (PHOG)	Self-similarity based on PHOG features	Amirshahi et al. (2012), Bosch et al. (2007), Braun et al. (2013)
	Self-similarity (CNN)	Self-similarity based on CNN features	Brachmann and Redies (2017b), Krizhevsky et al. (2012)
Feature distribution and entropy	Homogeneity	Shannon entropy of black pixel distribution across all subregions of a binarized image	Hübner and Fillinger (2016), Shannon (1948)
	Anisotropy (HOG)	Anisotropy of gradient orientations (HOG features)	Braun et al. (2013), Dalal and Triggs (2005)
	First-order and Second-order EOE	First-order and Second-order Edge-orientation entropy (Gabor filters)	Redies et al. (2017), Shannon (1948)
	Sparseness, Variability	CNN-based Sparseness and Variability (CNN feature variances)	Brachmann et al. (2017), Krizhevsky et al. (2012)

*Notes*. CNN, convolutional neural network; DCM, deviation of center-of-mass; EOE, edge-orientation entropy; (P)HOG, (pyramid of) histograms of oriented gradients; RMS, root mean square

We focus on quantitative properties of 2D static images, which have been studied more often than 3D or moving stimuli, such as architectural objects, sculpture, dance, and movies (Christensen & Calvo-Merino, 2013; Joye, 2007; Vukadinovic & Markovic, 2012). Moreover, we will put emphasis on image properties that are derived from the perceptual structure of large image areas or from the entire image. Examples are Fourier spectrum properties or the self-similarity of images (Aks & Sprott, 1996; Amirshahi et al., 2012; Graham & Field, 2007; Redies et al., 2007). Such image properties are of particular interest because many of the image qualities that bring about subjective aesthetic impressions, such as “composition”, “visual rightness” (Locher et al., 1999; Vissers & Wagemans, 2023), and “good Gestalt” (Arnheim, 1954), refer to global rather than to local image structure. For each image property, we will explain why it is relevant for aesthetics research and how it can be calculated. To contextualize the image properties, we will refer to some exemplary studies that illustrate their usage and their relation to subjective aesthetic ratings, such as liking, visual preference, beauty, or artistic value. A detailed review of this relation is beyond the scope of the present work.

For the Toolbox, we restrict our selection mostly to image properties that are known to us from our own research groups and for which we have the original code. Note that our coverage of image properties is not complete, and there are other properties studied in experimental aesthetics. We do not attempt to give an exhaustive or general overview of this field of research in the present work. Rather, we want to endow investigators with an understanding of how to calculate a subset of specific image properties and how to use them in aesthetics research. Another criterion for the inclusion of image properties in the Toolbox was that the properties reflect a wide spectrum of low- and mid-level image properties. We tried to exclude highly redundant properties as much as possible. Therefore, we believe that our choice of image properties (Table 1) is representative for the larger set and probably also includes the properties that are used most frequently.

Other code to calculate image properties used in visual aesthetics research is freely available online in various repositories. Examples are the toolbox by Mayer and Landwehr (2018), which computes four image properties related to processing fluency in the visual system (simplicity, symmetry, contrast, and self-similarity; Mayer & Landwehr, 2018). Walther et al. (2023) designed a set of computational tools to analyze mid-level visual properties, including local symmetry, contour properties, and perceptual grouping cues in real-world images. To study the relation between order, complexity, and aesthetic ratings, Van Geert and colleagues (2023) conceived a toolbox that allows researchers to create multi-element displays that vary qualitatively and quantitatively in different kinds and measures of order and complexity. Last but not least, Peng (2022) provided a tutorial on how to calculate image properties, such as color attributes and complexity, for research in the social sciences.

The advantage of the Aesthetics Toolbox is that it is a coherent, open-access web application written in a single programming language, Python 3^1^. To our knowledge, it is the first toolbox that allows the calculation of image properties from multiple research groups (see Table 1) on a single web interface. The Toolbox can be used without any proprietary software license. It is open source, fully documented, tested, and maintained. All scripts in the toolbox are based on original code written by researchers in the field of aesthetics, ensuring that all image properties produce the same results as in the original studies for which they were developed. This approach ensures comparability of results between the original code and the Toolbox, but neglects more recent modifications and extensions of the code. In some cases, our approach generates inconsistencies, which are mentioned in the text.

The code and the calculation methods are documented in detail. This documentation allows researchers to replicate, compare, and extend the results of aesthetics research. The Toolbox has a browser-based graphical user interface (GUI) build with *streamlit* (https://streamlit.io) for easy usage and does not require programming knowledge. Finally, other research groups are invited to expand the Toolbox by contributing their own image properties and techniques to compute them. Thus, we hope the Toolbox will become a valuable means to investigate quantitative image properties in aesthetics research.

### Quantitative image properties

This section provides a general introduction to the image properties that can be calculated with the Toolbox (Table 1). Additional computational details are documented online and in the code.^1^ The content and text of the present description of the Toolbox partially overlap with the online documentation. We restricted our Toolbox to image properties, for which original code was available. For each image property, our Python code is based on the latest available version of the respective code. We confirmed that the code implemented in our Toolbox yields results identical to those from the original version of the code supplied to us, except for properties where this was not possible for technical reasons (i.e., HOG complexity and anisotropy). We will describe the image properties in the following sections, proceeding from relatively simple properties to more complex ones. Image properties are grouped more or less according to which conceptual type of property they reflect (e.g., complexity, color, balance, or self-similarity) rather than by which method they are calculated (e.g., properties derived from convolutional neural networks, CNNs). As a general convention, we capitalize the first word in the names of the image properties that are calculated with the Aesthetics Toolbox.

**Fig. 1:**
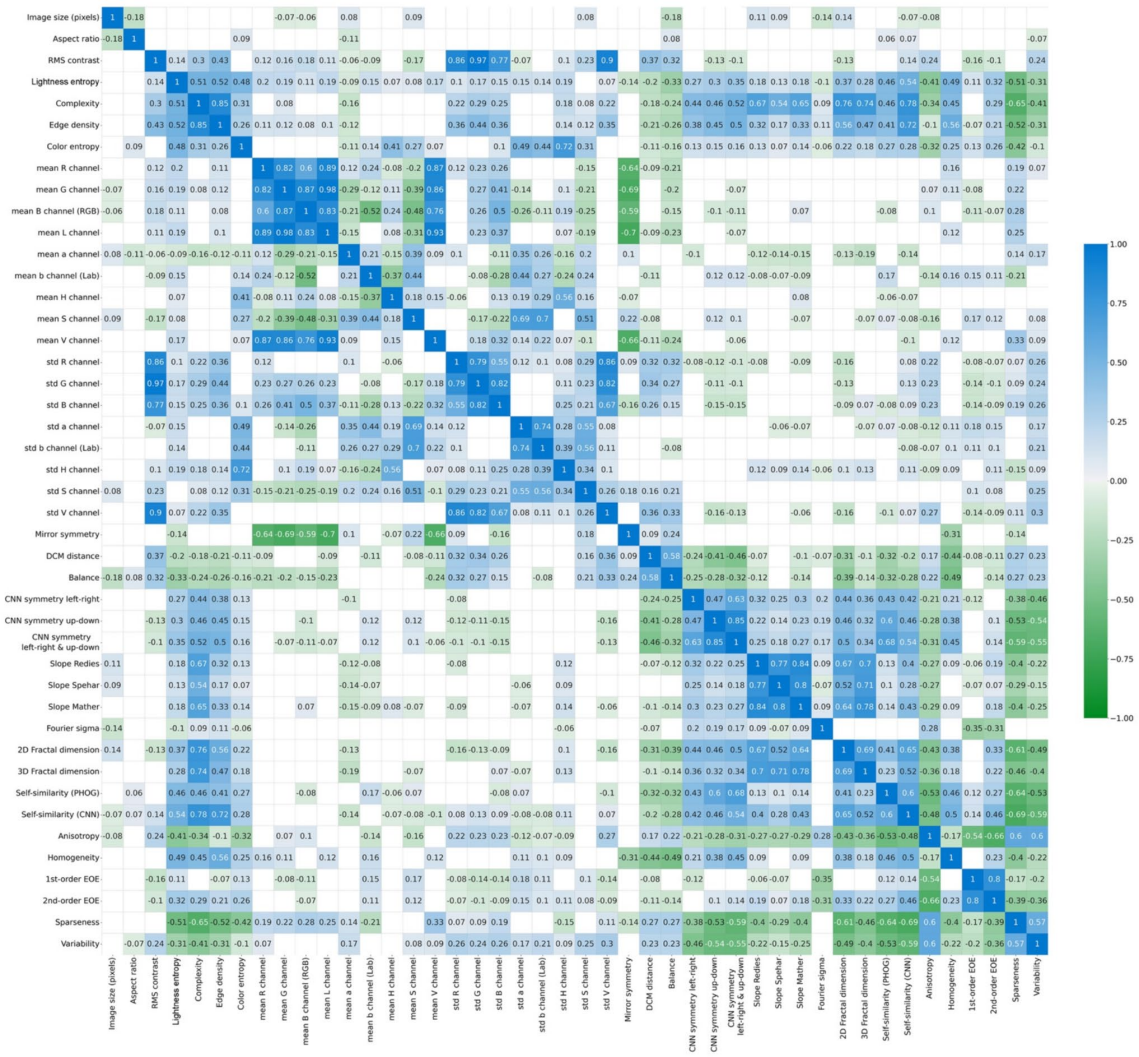
Spearman’s coefficients of correlation ρ for image properties of the AVA dataset. *Notes.* Image properties were calculated with the Toolbox for 1000 randomly selected images from the AVA dataset. The colored shadings represent the strength of the Spearman’s correlation coefficients ρ(for color coding, see the right-hand side of the panel). Significant correlations only are shown (*p* < 0.05). CNN, convolutional neural network; EOE, edge-orientation entropy; std, standard deviation; PHOG, pyramid histogram of oriented gradients

Figure 1 displays a correlation matrix for all 43 image properties calculated with the Toolbox for 1000 randomly selected photographs of diverse subject matters from the AVA dataset of artworks (Murray et al., 2012). For this dataset, we also show scatter plots for selected image properties in the present work. Corresponding data for 1629 traditional Western paintings from the JenAesthetics dataset (Amirshahi et al., 2015) and for a set of 1000 random-phase images (Branka Spehar, unpublished data) are provided in the GitHub repository of the project.^1^

### Image dimensions and scaling

#### Image size

Calculating image size may seem relatively straightforward at first sight. Nevertheless, there are several different ways to calculate this property, including the product of height and width, the diagonal, or the maximum, average, or minimum of height and width. For each method, there are advantages and limitations, and extremes can be considered where the objective (measured) values deviate strongly from the subjective impression of image size. We follow Datta et al. (2006) in the present study and define Image size as the sum of width and height as the default measure. Some of the other ways to calculate image size are also implemented in the Toolbox.

Importantly, the calculated values of several other image properties depend on image size. Obvious examples are the measures that reflect image complexity. Other properties are less affected, such as the means of the color channels and their standard deviations. Therefore, it is advisable to use the same or a similar image size to calculate image properties, especially when absolute values of the same image property are compared between images. We recommend a minimum length of 1024 pixels on the longer side of each image in order to avoid upscaling during pre-processing of images (for details on image pre-processing, see the documentation for individual image properties).

Particular attention should be given also to the experimental conditions that affect image size in psychophysical experiments, e.g., in rating studies (Clarke et al., 1984). In general, it is the size of the image on the retina that matters in such experiments, not their physical size on the screen or the image size expressed in pixels. Consequently, viewing distance must be taken into account when image size is compared between psychological studies. In support of this notion, Carbon (2017) demonstrated a strong positive relationship between canvas size and preferred viewing distance when beholders freely viewed works by the artist Gerhard Richter in a museum context. The role of image size was also studied by Munar et al. (2023) who found that participants in a museum context chose to view curved abstract paintings by the artist Robert Pepperell from a closer distance (i.e., with larger image size on the retina) than angular ones.

#### Aspect ratio

The methods for calculating aspect ratio lack consistency in the literature. The aspect ratio has been calculated as either the height-to-width ratio (e.g., see Mallon et al., 2014) or the width-to-height ratio (e.g., see Datta et al., 2006; Iigaya et al., 2021; Li et al., 2006). In the Toolbox, we follow the latter metric, which is also the convention used for specifying display format in commercial settings. Because the two measures are the inverse of each other, they correlate negatively.

Care should be taken to avoid changing the aspect ratio for the calculation of image properties. Changing the aspect ratio usually involves a change in image size. Moreover, changing the aspect ratio, e.g., to obtain uniformly square images, will necessarily affect the aesthetic appeal of images because the depicted objects or scenes may appear distorted to the observer.

The aspect ratio was one of the first quantitative image properties to be studied in experimental aesthetics. Based on his psychophysical experiments with simple geometrical forms, Fechner (1876) claimed that rectangles with a golden aspect ratio (length of longer side over shorter side of about 1.618) are aesthetically more pleasing to human observers than other proportions. Many Western artists and architects are believed to have realized the golden ratio in their works since the time of the Ancient Greeks. However, results from carefully controlled modern studies do not support the general aesthetic superiority of the golden ratio (McManus et al., 2010; Plug, 1980). Nevertheless, many of the media that are used in contemporary psychological studies to display visual stimuli, such as monitors and mobile phones, have screens with standardized size ratios that may have a direct or indirect effect on aesthetic liking (Tractinsky et al., 2013).

### Contrast and complexity

#### Root mean square (RMS) contrast

Contrast is a commonly studied feature in aesthetics research. In the Toolbox, contrast is calculated as the root mean square (RMS) Contrast, which corresponds to the standard deviation of pixel values in the Lightness (L) channel of the L*a*b* (or CIELAB) color space (Peli, 1990). Contrast is higher if pixel values are distributed over a larger range of Lightness values. Various other measures of contrast that are not included in the Toolbox, have been proposed (e.g., by Li & Chen, 2009; Luo & Tang, 2008; Schifanella et al., 2015; Tong et al., 2004).

Several studies demonstrated that images of high contrast are generally preferred over images of low contrast (e.g., see Tinio et al., 2011; van Dongen & Zijlmans, 2017).

#### Lightness entropy

Entropy is a concept used in various fields, ranging from thermodynamics to information theory. In general, low entropy is associated with highly organized, ordered structures, and the transition towards less organized states increases entropy. In information theory, Shannon entropy is a measure of the uncertainty of information content in a message source (Shannon, 1948). When applied to images, Shannon entropy measures the degree to which an image feature varies in a random fashion; it is inversely related to the notion of spatial redundancy (Kersten, 1987; Mather, 2018). A common way of expressing entropy of an image is with respect to the range of states or values that local samples of an image, such as pixels, can possess. These states are often summarized in histograms of the respective image property. The Toolbox includes measures for the entropy of (a) pixel intensities (this section), (b) color hue (section "Colorfulness"), (c) the spatial distribution of pixel intensity (section "Homogeneity"), and (d) edge orientation (section "Edge-orientation entropy").

The Aesthetics Toolbox calculates the Shannon entropy of pixel intensity based on the Lightness (L) channel of the L*a*b* (or CIELAB) color space. This measure has been simply referred to as ‘entropy’ by other researchers (Iigaya et al., 2021; Mather, 2018; Sidhu et al., 2018). To avoid confusion, we refer to our measure as *Lightness entropy* in the present work. Lightness entropy is a measure of the randomness, or uncertainty, of the pixel Lightness values in an image. If all pixel values occur with the same frequency in an image, the calculated Lightness entropy values are high. If particular pixel values occur more frequently than others, Lightness entropy assumes lower values. Sidhu et al. (2018) reported that the beauty of abstract paintings is rated higher when the entropy of pixel intensities values is higher.

#### Complexity

Image complexity can be broadly defined as the quantity and variety of information in an image. In the Toolbox, we include two relatively straightforward measures of complexity that have been used in visual aesthetics research before. The first measure is based on lightness and color gradients in an image and is here simply called Complexity (Braun et al., 2013). Histograms of lightness and color gradients (HOG features; Dalal & Triggs, 2005) are also used to calculate Anisotropy (see below, section “Anisotropy of gradient orientation”) and Self-similarity (see below, section “HOG-based Self-similarity”). The second measure is based on edge responses in Gabor-filtered images (here called Edge density; Mehrotra et al., 1992). Gabor-filtered images are also used to calculate Edge-orientation entropy (see below, section “Edge-orientation entropy [EOE]”).

##### Complexity (HOG method)

**Fig. 2:**
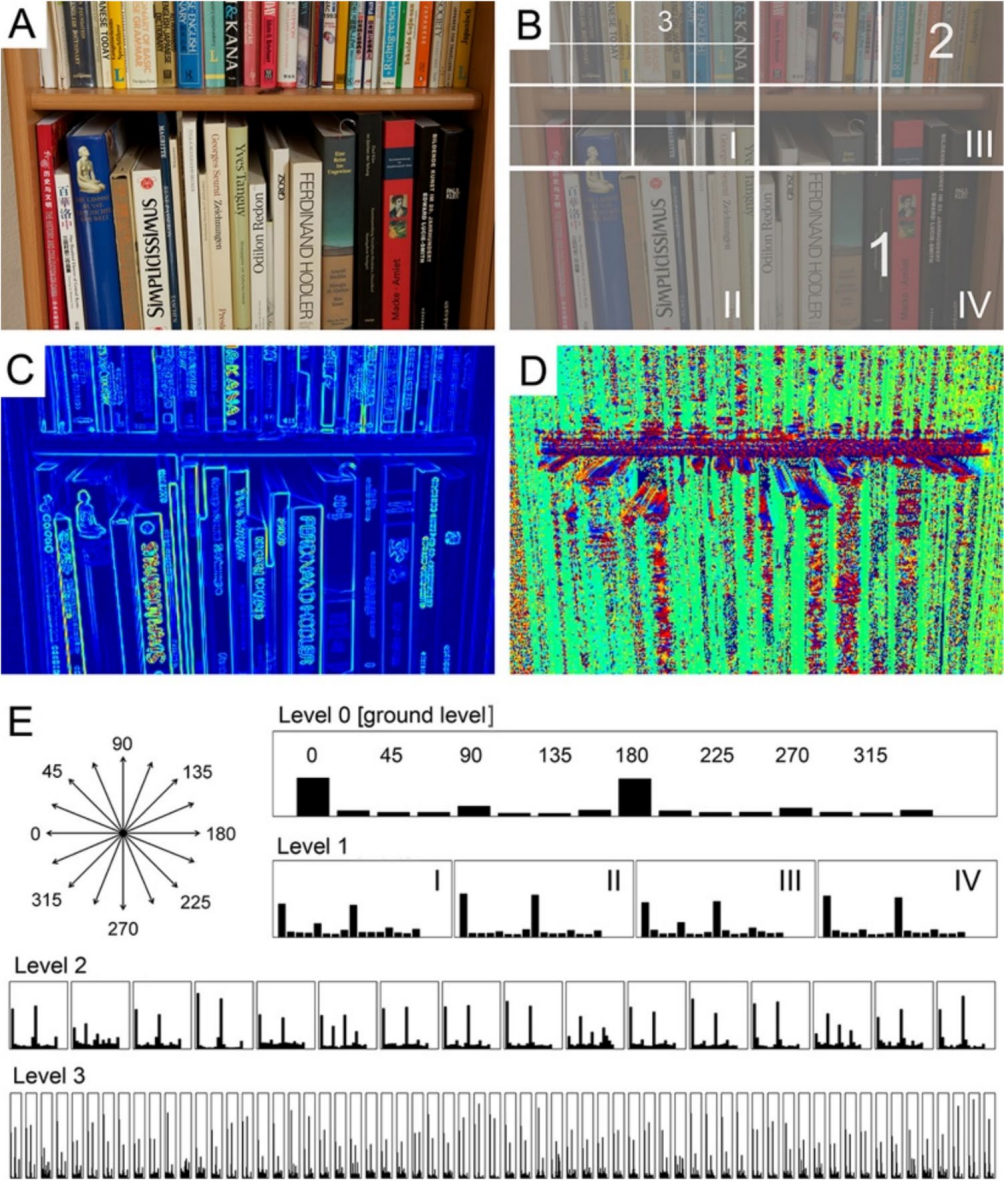
Method for calculating complexity, anisotropy and self-similarity by the (P-)HOG method. *Notes.* **A** Original photograph. **B** Schematic diagram of the subdivisions at different levels of spatial resolution (Levels 1–3). **C** Pseudo-colored gradient strength image. **D** Image of pseudo-colored orientations (e.g., *red* for horizontal orientations, and *green* for vertical orientations). **E** shows the HOG features (histograms of gradient orientations) at the different levels of spatial resolution (Levels 0–3). The orientations of the 16 bins used for calculating the HOG features are displayed at the upper left side of the panel. The Roman numbers I–IV in **B** and **E** denote the subsections at Level 1. For more details, see text. Reproduced from Braun et al. (2013) with permission

To calculate Complexity values with the Histogram of Oriented Gradients (HOG) method (for details, see appendix in Braun et al., 2013; Dalal & Triggs, 2005), each image is converted into the L*a*b* color space (for a sample image, see Fig. 2A), and then separated into its three channels. For each pixel, the highest value among the three pixel values of the channel images is selected and placed into a new combined so-called *gradient image* (Fig. 2C). Because gradients are generally stronger for the L* (lightness) channel than for the a* and b* channels, the lightness gradients generally dominate the combined gradient image. The mean pixel value over the combined gradient image is used as the measure of the Complexity of the image. A uniform original image with small changes in pixel values would result in a gradient image of low mean values, i.e., low Complexity, while an image with large changes would result in a gradient image of high mean values, i.e., high Complexity. The Complexity values calculated by this method depend on image size (Fig. 7 in Redies & Groß, 2013). As mentioned above, it is thus recommended to use a uniform input size for the images analyzed. The desired image size can be set in the parameter section of this image property in the Toolbox.

##### Edge density (Gabor filters)

As another measure of image complexity, we estimate the Edge density in Gabor-filtered images (Mehrotra et al., 1992), following the procedure described by Redies and colleagues (2017). In brief, the Toolbox starts by automatically converting the images to grayscale. Each input image is then reduced to a maximum of 120,000 pixels. This is the maximum number of pixels used in the original algorithm for computational reasons. It is used here also in the Toolbox version to ensure that results can be compared. Next, we apply a bank of 24 oriented Gabor filters with equally spaced orientation bins that cover one full rotation (360°) to extract oriented edge elements from each image. Gabor filters resemble receptive fields at low levels of the human visual system. Edge density is calculated as the sum of all edge responses in each Gabor-filtered image. The resulting value reflects not only the density of edges in an image, but also edge strength (i.e., contrast).

Like other measures based on the number and strength of lightness gradients in an image (Forsythe et al., 2011), HOG-based Complexity and Edge density correlate with perceived (subjective) complexity.

**Fig. 3:**
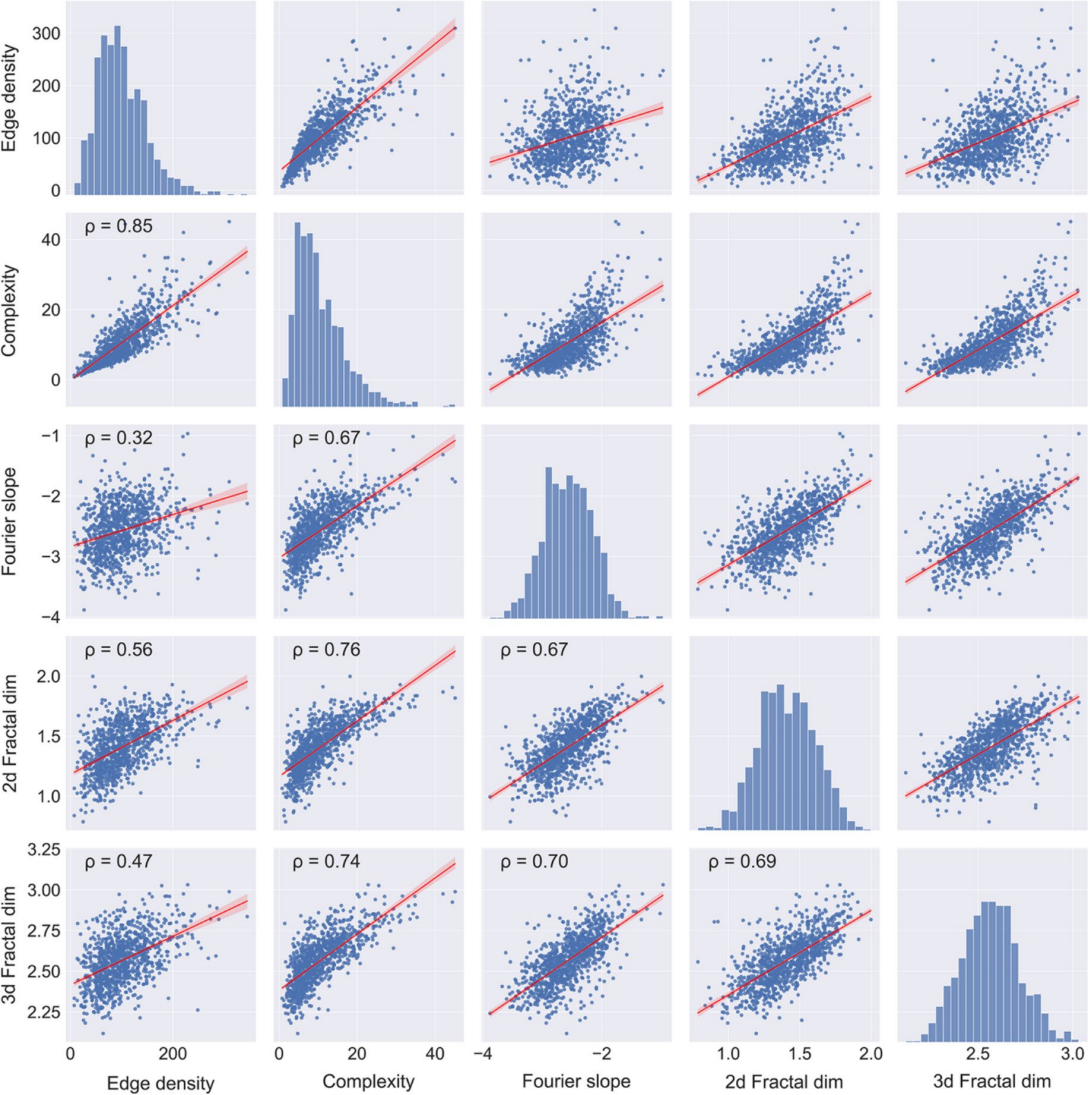
Scatter plots for the image properties that relate to image complexity. *Notes.* Results for 1000 images randomly selected from the AVA dataset are shown. All correlations are significant (*p* < 0.05). The *red line* represents the result from linear regression with the 95% confidence interval indicated by *red shading*. Frequency histograms for each image property are shown along the diagonal axis. 2D Fractal dim, 2D Fractal dimension *D*; 3D Fractal dim, 3D Fractal dimension *D*; ρ, Spearman’s coefficient of correlation

The Toolbox contains two additional measures that relate to image complexity (Bies et al., 2016b; Van Geert & Wagemans, 2020, 2021), i.e., the (2D and 3D) Fractal dimension *D* (Bies et al., 2016a; Mather, 2018; Spehar et al., 2016; Taylor et al., 1999) and the Fourier slope (Burton & Moorhead, 1987; Graham & Field, 2007; Redies et al., 2007; Spehar & Taylor, 2013; Tolhurst et al., 1992). Figure 3 displays scatter plots for pairwise combinations of the four complexity-related image properties in the Toolbox. Spearman’s coefficients of correlation $\rho$ range from 0.32 for Edge density versus Fourier Slope, to 0.85 for Edge density versus Complexity. There are several other ways to calculate image complexity (for reviews, see Bies et al., 2016b; Forsythe et al., 2011; McCormack & Gambardella, 2022; Nath et al., 2024; Peng, 2022; Rigau et al., 2008; Van Geert & Wagemans, 2020, 2021).

On average, observers prefer visual stimuli of intermediate visual complexity (Berlyne, 1970; Taylor et al., 2005b; Wundt, 1874), but there are large differences between individuals in which degree of complexity they prefer (Aitken, 1974; Bies et al., 2016a; Güclütürk et al., 2016; Spehar et al., 2016; Van Geert & Wagemans, 2021; Vissers et al., 2020).

### Color

Color is a pivotal feature of artworks and images in general (Albers et al., 2020; Bekhtereva & Muller, 2017; Li & Chen, 2009; Nakauchi et al., 2022; Nascimento et al., 2017; Palmer et al., 2013; Peng, 2022). Montagner et al. (2016) demonstrated that, for a set of 44 paintings of diverse subject matter, the color palettes used by the artists seem to be biased toward the yellow-red range of the spectrum. There is converging evidence that observers prefer a chromatic composition that is close to the original color composition of artworks, compared to versions with hue-rotated color gamuts (Nascimento et al., 2017, 2021; Albers et al., 2020; Altmann et al., 2021). Furthermore, observers perceive the original color compositions as more colorful (Altmann et al., 2021) and more natural (Nascimento et al., 2021) than hue-rotated versions. Nakauchi et al. (2022) showed that the color spectrum of artworks is not necessarily similar to that of natural scenes.

#### Channel means and channel standard deviations

To describe the color gamut of images, we capture color information in three color models (RGB, HSV, and L*a*b* [CIELAB]) that have been widely used in aesthetics research (Datta et al., 2006; Geller et al., 2022; Iigaya et al., 2021; Li & Chen, 2009; Li et al., 2006; Mallon et al., 2014; Nakauchi et al., 2022; Peng, 2022; Schifanella et al., 2015; Thieleking et al., 2020).

The RGB color space is one of the most popular ones; it is based on the primary colors of light and comprises a red (R) channel, a green (G) channel, and a blue (B) channel. There are different versions of this color space (e.g., sRBG and Adobe RGB). Differences in the color models used can have a large effect on the calculated image properties, even if an image displayed on a screen does not differ subjectively for the human eye between the various color models. A perceptually more intuitive representation of the RGB color space is the HSV model (H, hue; S, saturation; and V, value).

The L*a*b* color model describes a perceptually uniform space that has a lightness or intensity (L*) channel and two color-opponent channels, a* (green-magenta channel) and b* (blue-yellow channel), where positive values represent magenta and yellow color, respectively. The Toolbox calculates means and standard deviations for each channel of the three-color models.

#### Color entropy

As a complement to the above color values, we calculate the Shannon entropy of the Hue channel of the HSV color space to capture the colorfulness of an image (Color entropy; or HSV[H] entropy; see Geller et al., 2022). This measure shows high values if an image displays many color hues with about equal frequency across the entire range of hues, regardless of which colors these are in detail. An image with only a single hue would have a very low Shannon entropy in the Hue channel. Geller et al. (2022) showed that Color entropy can be positively or negatively related to aesthetic ratings, respectively, depending on which aesthetic dimension is rated (e.g., harmonious versus interesting).

### Balance and symmetry

Balance is a general attribute that reflects how well pictorial elements are arranged in the composition of an image and thereby contribute to its aesthetic appeal (Arnheim, 1954; Damiano et al., 2023; Hübner & Fillinger, 2016; McManus et al., 2011). To explain balance, Arnheim (1954) hypothesized that each rectangular frame possesses a field of invisible forces. The center of the framed image possesses the strongest attraction. The center is followed by its corners, the horizontal and vertical axis and then the diagonal axes. According to this model, every element placed in an image is pulled by all the invisible forces stemming from the pixelwise structure and, additionally, by all other pictorial elements in the image, thus creating an inner tension (Hübner, 2022; Hübner & Fillinger, 2016; McManus et al., 2011).

Symmetry is a special case of balance where an image can be divided into parts that are invariant under geometric transformations, such as reflection, rotation, or translation. (Liu et al., 2013). The human brain is particularly sensitive to symmetry (Bertamini et al., 2018; Jacobsen & Höfel, 2003). It is generally believed that symmetry is an important and universal basis of visual preference (for a review, see Bode et al., 2017; Damiano et al., 2023). However, the universal role of symmetry as an ‘aesthetic primitive’ (Latto, 1995) has been contested (Leder et al., 2019).

**Fig. 4:**
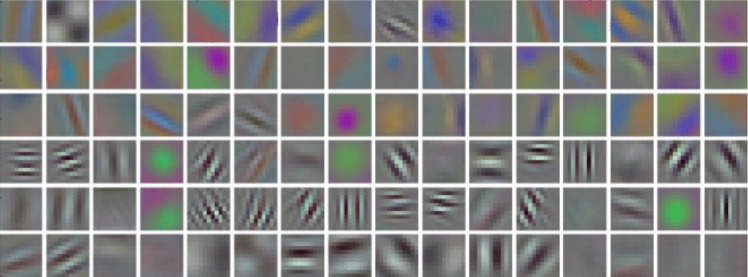
The 96 features on the first convolutional layer (conv1) of a CNN. *Notes*. The 96 filters are from an AlexNet network (Krizhevsky et al., 2012). They respond to luminance edges of different orientations and at different spatial frequencies, as well as to color-opponent gradients. Reproduced from Brachmann et al. (2017) with permission

**Fig. 5:**
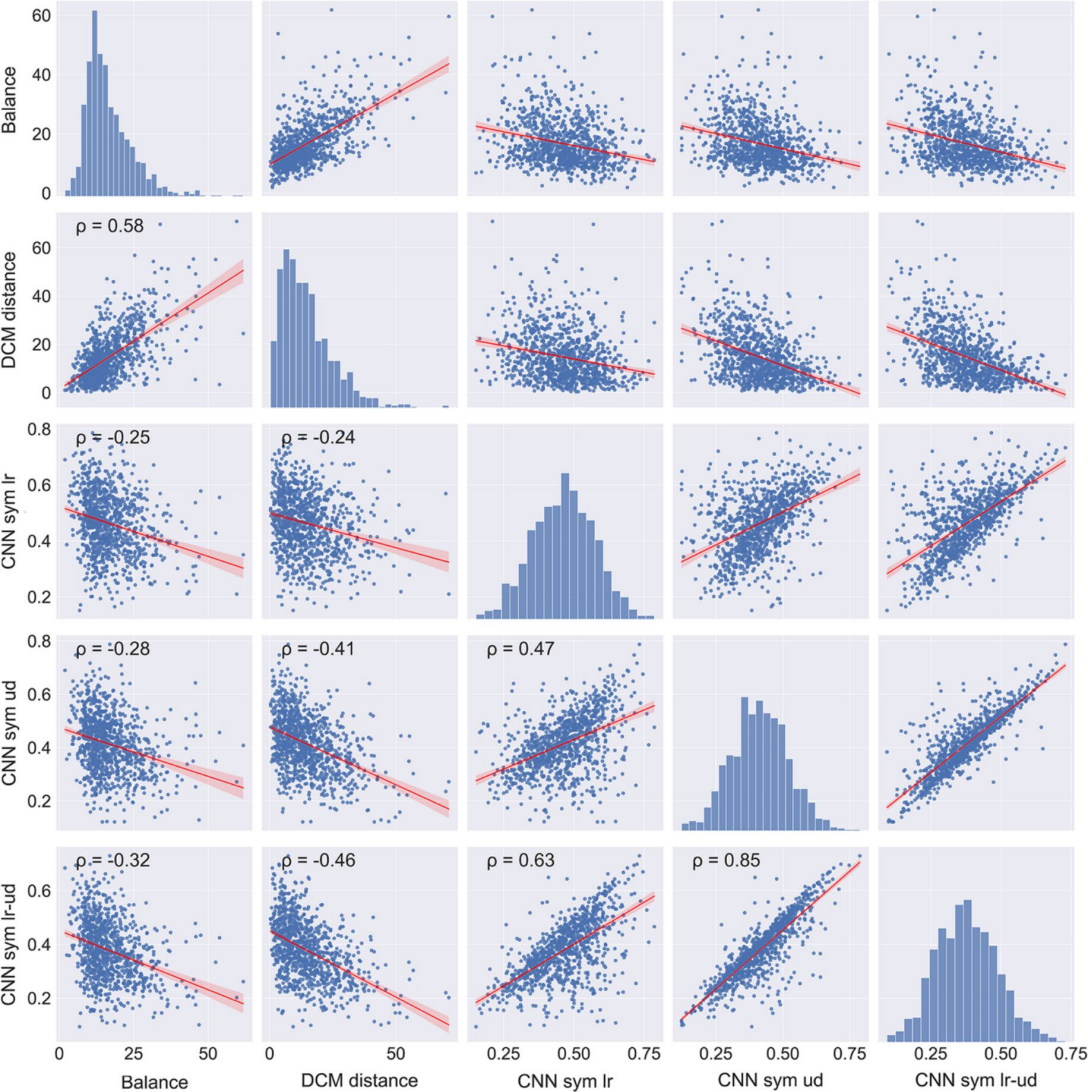
Scatter plots for the image properties that relate to balance and symmetry. *Notes.* Results for 1000 images randomly selected from the AVA dataset are shown. All correlations are significant (*p* < 0.05). The *red line* represents the result from linear regression with the 95% confidence interval indicated by *red shading*. Frequency histograms for each image property are shown along the diagonal axis. CNN sym lr, CNN-feature based left-right symmetry; CNN sym lr-ud, combined CNN-feature based left-right and up-down symmetry; CNN sym ud, CNN-feature based up-down symmetry; ρ, Spearman’s coefficient of correlation

Related to these ideas, the Toolbox includes three different pixel-based ways to calculate geometric Balance and Symmetry, as proposed by Hübner and Fillinger (2016). In addition, we describe a way to compute mirror symmetry based on features from low layers of a deep (convolutional) neural network (CNN) (Brachmann & Redies, 2016). These features are akin to filters in the early visual cortex (Fig. 4) and are thus more physiological than purely geometrical algorithms (see section “CNN feature-based Symmetry”). Figure 5 shows scatter plots for some of the image properties that reflect various aspects of symmetry in the Toolbox.

#### Pixel-based metrics

##### Balance

**Fig. 6:**
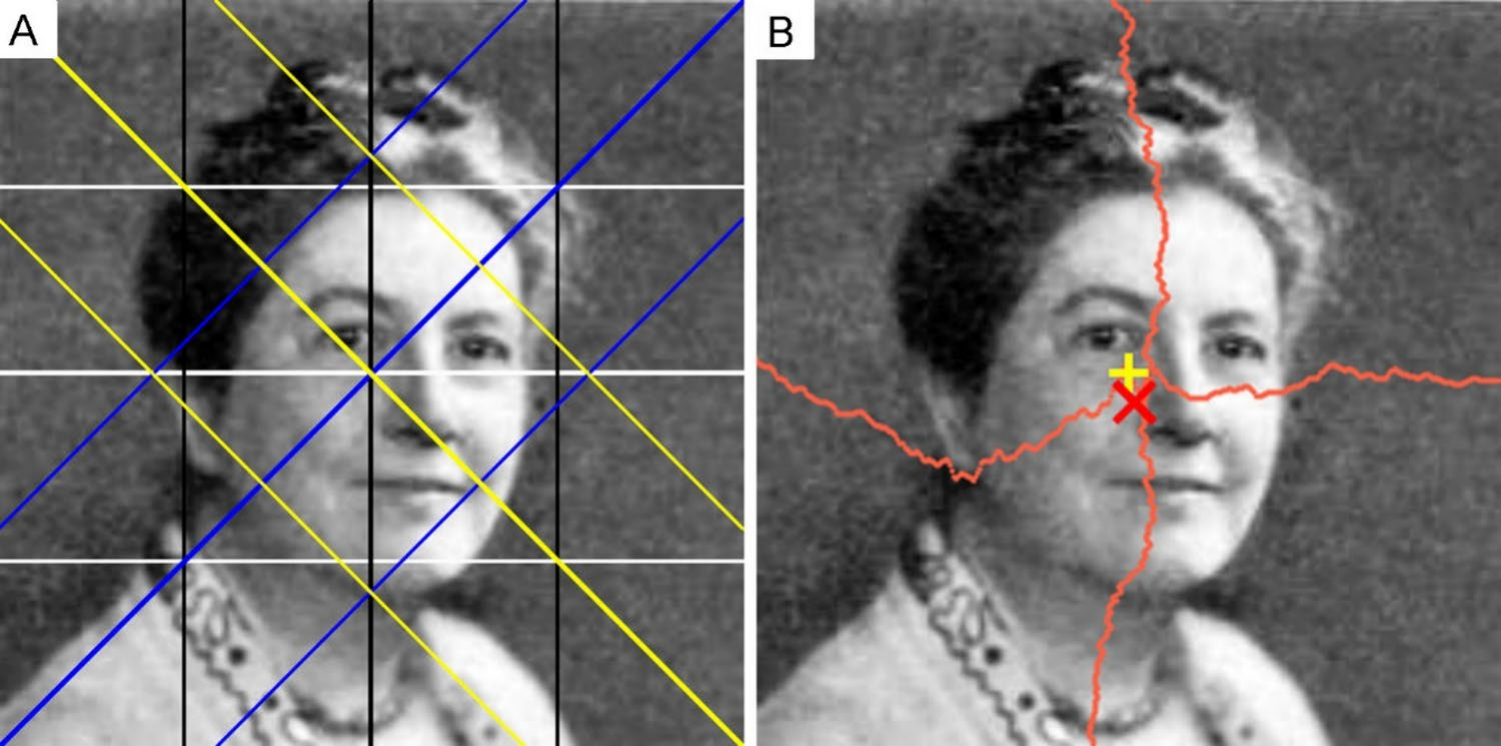
Schematic diagram for measuring the Balance score and the DCM score. *Notes.* **A** The different axes and lines used for computing the Balance score (Wilson & Chatterjee (2005); see text for details). The image underneath shows the portrait of Ethel Puffer (1872–1950, from a 1995 publication) who was one of the first psychologists to experimentally study balance (Puffer, 1903; Hübner and Thömmes, 2019). The Balance score of the image is 7.31 (*bright* pixels are assumed to be heavier), which indicates an excellent balance. **B** This picture shows the center of mass for each row and column in the image (*bright* pixels are assumed to be heavier). The *red X* marks the center of mass of the entire image and the *yellow cross* marks the geometrical center of the image. The DCM score is the distance between the two centers, expressed in percent of the maximum possible distance. The score is 7.21 in this case. Adapted from Wikimedia Commons (https://en.wikipedia.org/wiki/Ethel_Dench_Puffer_Howes; in the public domain)

Wilson and Chatterjee (2005) proposed an objective measure of balance, which they applied to black-and-white images composed of simple black geometric shapes on a white background. Their measure is based on a generalized concept of symmetry. The method calculates the absolute difference in black pixels for each of eight pairs of equally sized areas, which are defined based on the four main axes (horizontal, vertical, major diagonal, minor diagonal) and by pairs of lines parallel to each axis that divide the image into inner and corresponding two outer areas, respectively (see Fig. 6A). Each of the eight differences is then divided by the total sum of black pixels in the respective pair and multiplied by 100. The mean of the resulting percentage values is called “Balance score”. The closer its value is to 0%, the higher the balance. Wilson and Chatterjee (2005) showed that this objective measure of balance correlates highly with subjective preferences. The algorithm in the Toolbox for computing the Balance score also works on grayscale images like the one in Fig. 6. In this case, the differences are calculated between the summed gray levels in the two areas compared. If dark pixels are assumed to be ‘heavier’ than brighter pixels, then the highest possible gray level minus the actual gray levels must be summed.

##### Deviation of the center of mass (DCM)

A different measure of balance is derived from the concept of center of mass (McManus et al., 2011). Briefly, if the center of “perceptual mass” of all pixels in an image (depending on the perceptual weight and positioning of the respective element) is close to the geometric center of the image, then the image is fully balanced (Arnheim, 1954; Hübner & Fillinger, 2016; McManus et al., 2011). Using this approach, Hübner and Fillinger (2016) studied black-and-white images of simple geometric patterns similar to those of Wilson and Chatterjee (2005). They assumed that the “perceptual mass” of a black pixel is 1, while that of a white pixel is 0. Based on this assumption, they computed the Euclidean distance of the center of perceptual mass to the geometric center of the image for each axis and expressed this distance as a percentage of the maximum possible distance from the geometric center of the given image (here called *DCM score*). For example, if the center of mass (red X in Fig. 6B) were located in a corner of the image, the DCM score would be 100. As the DCM score is expressed as a percentage, like the Balance score, the two measures are easily comparable (see the scores in the legend to Fig. 6). In a rating study, DCM scores correlated more strongly with subjective balance ratings than the Balance score, while the opposite was observed for ratings of subjective preference (Hübner & Fillinger, 2016). The Toolbox implements a version of the DCM score that also works on grayscale images, like the one in Fig. 6, and has been applied previously by Thömmes and Hübner (2018). In an experiment on the cropping of photographs, Abeln et al. (2015) showed that the DCM for visual saliency was smaller for cropped details compared to avoided details.

##### Mirror symmetry

Two different approaches have been taken to measure geometric symmetry. One line of research focuses on detecting symmetry and symmetry axes (e.g., Wagemans, 1995, 1997). Another line focuses on measuring the strength of symmetry in an image. Here, we will follow the latter line. Many researchers focus on one particular kind of symmetry, namely reflectional symmetry (‘mirror symmetry’). Hübner and Fillinger (2016) defined this measure as the mean symmetry around the four main axes of an image (vertical, horizontal, and orthogonal axes; see above). For quadratic images, a mean Mirror symmetry (MS) score is calculated from these four symmetries and expressed in percent. Higher values indicate more symmetrical images. For other (non-quadratic) rectangular images, the mean MS score is calculated for the vertical and horizontal axes only, which corresponds to the original version of the method (Hübner & Fillinger, 2016). In future versions of the Toolbox, diagonal symmetry could be considered as well.

#### CNN feature-based symmetry

Brachmann and Redies (2016) developed a measure of symmetry that is based on filter responses from the first layer of a convolutional neural network (CNN, AlexNet; Krizhevsky et al., 2012). The CNN was trained on ImageNet for object recognition. Both the original implementation and the toolbox version use the CNN weights provided by the Caffe library (Jia et al., 2014). The CNN features match responses of neurons in the early visual cortex of higher mammals (for reviews, see Bowers et al., 2022; Kriegeskorte, 2015; Lecun & Bengio, 1995; Lindsay, 2020; Rafegas & Vanrell, 2016) and capture features such as oriented edges, color-opponent blobs, and spatial frequency information (Fig. 4). These features are reminiscent of the independent components of natural scenes (Bell & Sejnowski, 1997; Hyvärinen & Hoyer, 2001) and also emerge during the learning of a sparse code for natural images (Olshausen & Field, 1996; for a review, see Simoncelli & Olshausen, 2001).

To measure higher-level symmetry with the Toolbox, we used first-layer (*conv1*) filter responses of the AlexNet (Fig. 4) to compute Left-right symmetry, Up-down symmetry, and a symmetry measure that combines all four directions (Left-right-up-down symmetry), according to the algorithm by Brachmann and Redies (2016). The authors showed that the CNN-based symmetry scores predict human perception of symmetry with high accuracy.

### Scale invariance and self-similarity

Natural scenes contain a high degree of regularity in their statistical structure, despite their considerable surface-level heterogeneity (Burton & Moorhead, 1987; Field, 1987; Ruderman, 1994; Tolhurst et al., 1992). Large subsets of artworks resemble natural scenes in this respect (Graham & Field, 2007; Graham & Redies, 2010; Mather, 2018; Redies et al., 2007; Taylor et al., 2005a). Because of this correspondence, natural scene statistics have been investigated in artworks and other types of aesthetically preferred images.

In natural environments, it is commonly observed that neighboring regions exhibit greater similarity in their spatial characteristics – e.g., textures, colors, orientation – compared to more distant regions. Structures such as branches, ferns, or diverse growth patterns exhibit recurring, scale-invariant, patterns at multiple levels of magnification (Mandelbrot, 1983). These consistent patterns are closely connected to the concept of self-similarity. On a general level, scale invariance is a feature of systems, statistics, or objects that do not change properties and remain similar if their scale changes by a certain amount, i.e., if one zooms in and out of an image. Self-similarity is a property in which a form is made up of parts similar to the whole or to one another. The discovery that the abstract drip paintings by Jackson Pollock have a scale-invariant (fractal) structure (Taylor, 2002) was one of the first examples of an objective and complex image property measured in artworks.

The scale-invariant or self-similar properties can be quantified by using a number of distinct scaling parameters. Two widely used measures are the slope of the Fourier amplitude spectrum (α) and the fractal dimension (*D*). The two measures have been related to each other both mathematically (Graham & Field, 2008; Knill et al., 1990) and empirically (Bies et al., 2016b). Different methods to calculate these two parameters are implemented in the Toolbox. In addition, we describe two further methods to calculate self-similarity by using features that more closely resemble neural responses in the early mammalian visual system (see section "Measures of self-similarity inspired by vision models [PHOG and CNN]").

#### Fourier spectrum slope

**Fig. 7:**
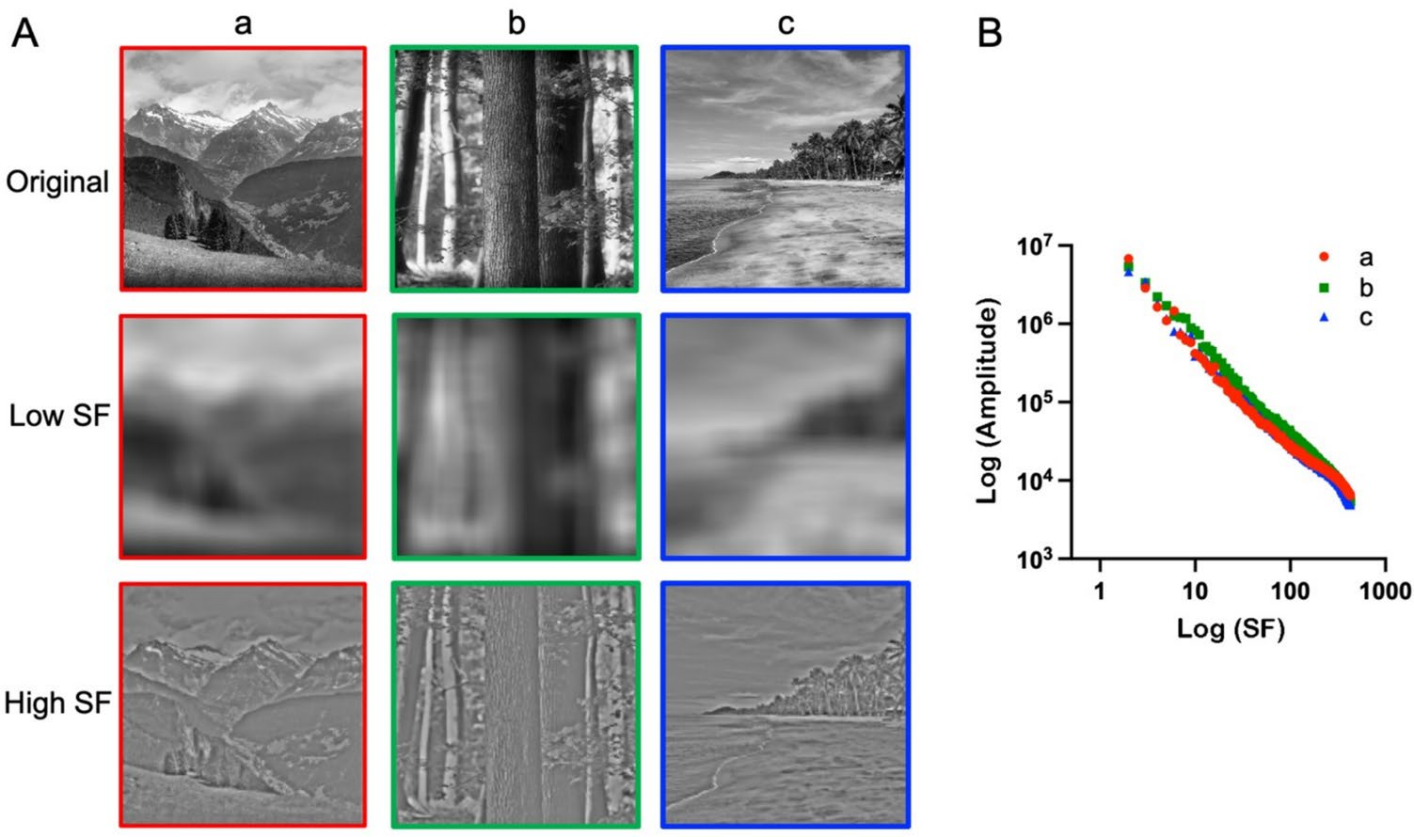
Fourier spectrum slope of three natural sample images *Notes.* **A** Original images of natural scenes (*top row*) and each image filtered for low spatial frequencies (*middle row*) and high spatial frequencies (*bottom row*), respectively. **B** Log-log plots of the Fourier amplitude spectra of the original images. SF, spatial frequency. Adapted from Viengkham et al. (2022) with permission

A common way to represent the spatial distance-dependent regularities regarding the intensity variations across natural scenes and other types of images is through the shape of their spatial frequency amplitude (Fourier) spectra. Figure 7A depicts the original versions and the spatial frequency-filtered versions of three different natural scenes. When the initial scenes are broken down into distinct spatial frequency components – depicted in the middle row (low spatial frequency-filtered images) and bottom row (high spatial frequency-filtered images) – it becomes evident that the relative amplitude of intensity variations is inversely correlated with the spatial frequency content of these images. This relationship is illustrated in the right panel of Fig. 7B. Amplitude decreases according to a power law, which shows up as a linear relationship in a log-log plot for most natural objects and scenes. This linear decrease is believed to indicate the scale invariance of natural scenes, suggesting that similar spatial structure can be observed as we zoom in or out across the coarse or fine spatial scales (i.e., low and high spatial frequencies, respectively).

The power-law relationship between the amplitude and spatial frequency is characterized by the amplitude spectrum slope α which, on average, ranges from – 0.8 to – 1.5 (peaking at – 1.2) for images of natural scenes. If power is plotted instead of amplitude in log-log plots, the average peak of the power spectrum slope is about – 2.4 (i.e., equal to twice the amplitude spectrum slope). Several types of aesthetically preferred images, including traditional artworks, have slope values similar to those of natural scenes (Graham & Field, 2007; Graham & Redies, 2010; Koch et al., 2010; Mather, 2018; Redies et al., 2007).

Fourier slope can also be interpreted as a measure of complexity (Spehar et al., 2016; Van Geert & Wagemans, 2020, 2021) because shallower slopes (i.e., less negative slope values) are indicative of more fine detail in an image. Moreover, the Fourier spectra of images contain cues that can be used to objectively discriminate between angular and curvilinear stimuli (Watier, 2018). Curvilinear stimuli, which are generally preferred over angular stimuli (Bar & Neta, 2006; Gómez-Puerto et al., 2016), possess a lower number of peaks in plots that sum up the magnitudes over frequencies along specific orientations of the Fourier spectrum (Watier, 2024). In a similar vein, visually preferred images, such as artworks and graphic novels, are more homogeneous across the orientations of the Fourier spectrum than other types of stimuli, such as non-artistic photographs of objects or natural scenes (Koch et al., 2010). These findings suggest that a Fourier spectrum that is homogeneous (i.e., isotropic) across orientations may be indicative of visual preference of certain types of images. Note that the information about the radial distribution of the Fourier amplitude is an attribute of the 2D Fourier spectrum that is lost during radial averaging when the Fourier spectrum Slope is calculated.

Fourier spectrum slopes have been investigated by many research groups (e.g., see Burton & Moorhead, 1987; Field, 1987; Graham & Field, 2007; Mather, 2014; O’Hare & Hibbard, 2011; Redies et al., 2007; Ruderman, 1994; Spehar & Taylor, 2013; Spehar et al., 2016; Tolhurst et al., 1992). There are several implementations of the algorithm that are similar but differ in important details. The general approach is to first Fourier transform the grayscale version of the image. In a log-log plot of the radially averaged Fourier spectrum (Fig. 7B), the decrease in spectral power (or amplitude) is then approximated by a linear regression. The slope of this regression line is the Fourier spectrum Slope α.

Specific implementations of these Fourier measures can vary in details of image pre-processing, the choice of the spectrum plot (amplitude or power), and which frequencies are excluded from the fitting because they represent noise or are prone to artifacts, such as uneven illumination, rectangular sampling, raster screen or noise in the images. Seemingly minor differences in implementation details can cause substantial deviations in absolute terms and in the between-group correlations of the Slope values.

In the Toolbox, we include three different versions for calculating the Fourier Slope. They are the methods by Branka Spehar and colleagues (Isherwood et al., 2021; Spehar & Taylor, 2013), by Christoph Redies’ group (Koch et al., 2010; Redies et al., 2007), and by George Mather (Mather, 2014). Differences between these versions are listed in Table 2.

**Table 2:** Characteristics of three methods to calculate the slope values of the Fourier (amplitude or power) spectrum

	Code provided by		
	B. Spehar’s Lab	C. Redies‘ Lab	G. Mather
Pixel values	8-bit grayscale	8-bit grayscale	L (Lightness) channel of the MATLAB CIELAB color space
Image pre-processing and image format	Center crop to largest square with power of 2 (unless square already)	Padding images to square with mean gray value and resizing to 1024 × 1024 pixels	Center crop to largest square with power of 2 and resizing to 1024 × 1024 pixels
Fourier spectrum	Amplitude	Power	Amplitude
Frequency omitted from fitting	Frequencies with Cook’s distance > *n*/4	Frequencies below 10 and above 256 cycles/image	Lowest quartile and highest quartile of frequencies
Binning of frequencies	None	Binning of data points at regular intervals in log-log plot	None
Reference(s)	Isherwood et al. (2021)	Redies et al. (2007); Koch et al. (2010)	Mather (2014)

*Notes. n* corresponds to half of the image width

**Fig. 8:**
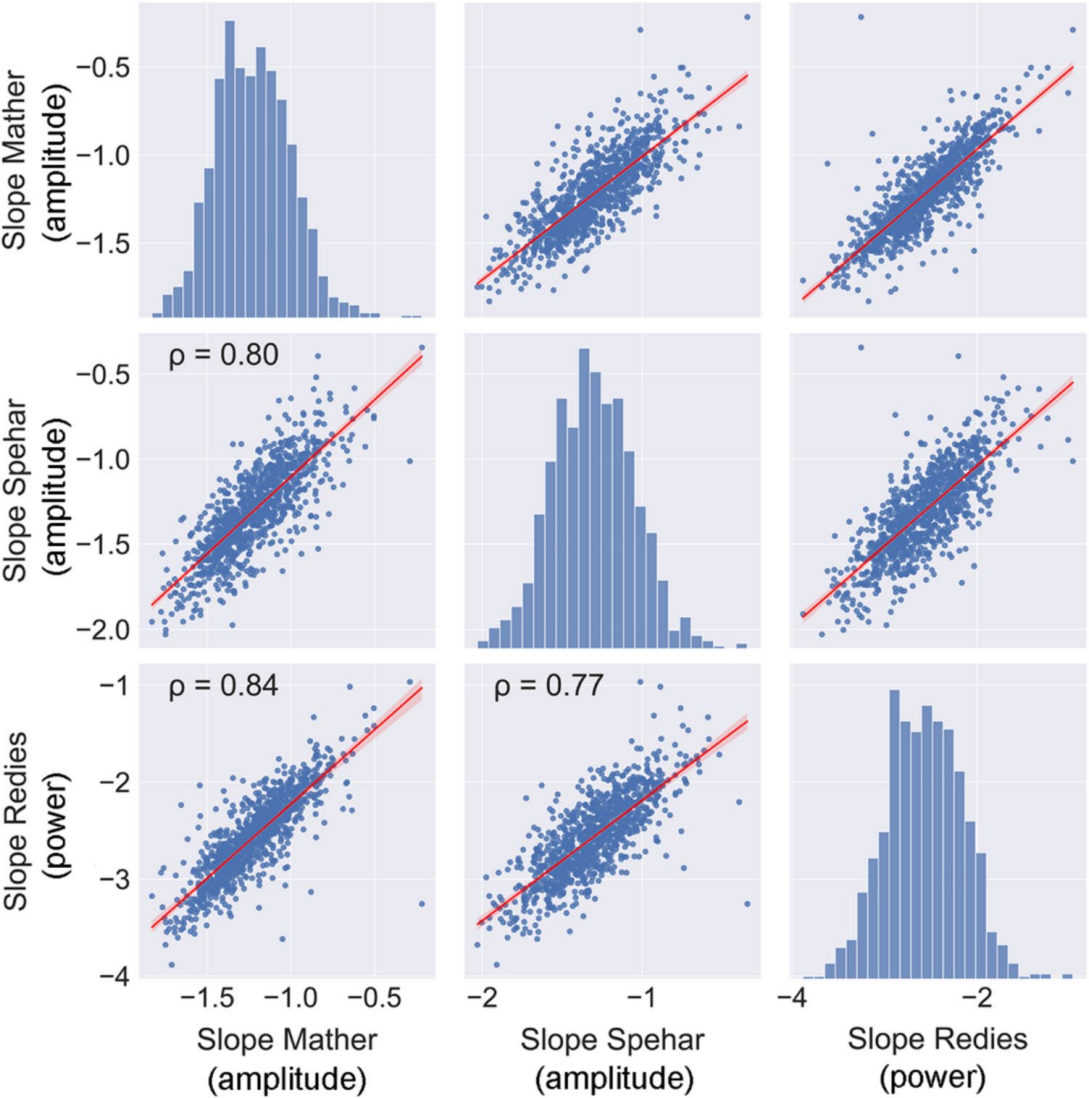
Scatter plots for the methods to calculate Fourier spectrum slope. *Notes.* Results for 1000 images randomly selected from the AVA dataset are shown. All correlations are significant (*p* < 0.05). The *red line* represents the result from linear regression with the 95% confidence interval indicated by *red shading*. Frequency histograms for each image property are shown along the diagonal axis. ρ, Spearman’s coefficient of correlation

To assess how similar the Slopes values are between methods, we determined the Spearman’s coefficient of correlation for three sets of images: (a) 1000 randomly selected images from the AVA dataset (Murray et al., 2012), (b) the JenAesthetics dataset of 1629 traditional Western paintings (Amirshahi et al., 2015), and (c) 1000 artificially generated random-phase images (Branka Spehar, unpublished data). Spearman’s coefficients of the Slope values calculated with the three different methods were 0.77–0.84 for the AVA dataset (Fig. 8), 0.67–0.88 for the JenAesthetics dataset, and 0.98–1.00 for the random-phase dataset. Scatter plots for the JenAesthetics dataset and the random-phase dataset can be found at the GitHub site of the present work.^1^ Slope values obtained with the three methods are therefore not fully comparable between methods for all data sets. However, for the artificially generated random-phase images, the results correlate almost perfectly. Because it is hardly possible to decide which method is better from a theoretical point of view, we included all three versions in the Toolbox so that users can take their choice.

Ambiguities in calculating the image properties, as exemplified by the Fourier Slope, underline the need to exchange and share algorithms if the results from different studies are to be compared. Ideally, a standardized set of algorithms should be used by researchers in the field. The reimplementation of algorithms based on vague or incomplete descriptions in papers should be discouraged.

#### Fourier spectrum sigma

**Fig. 9:**
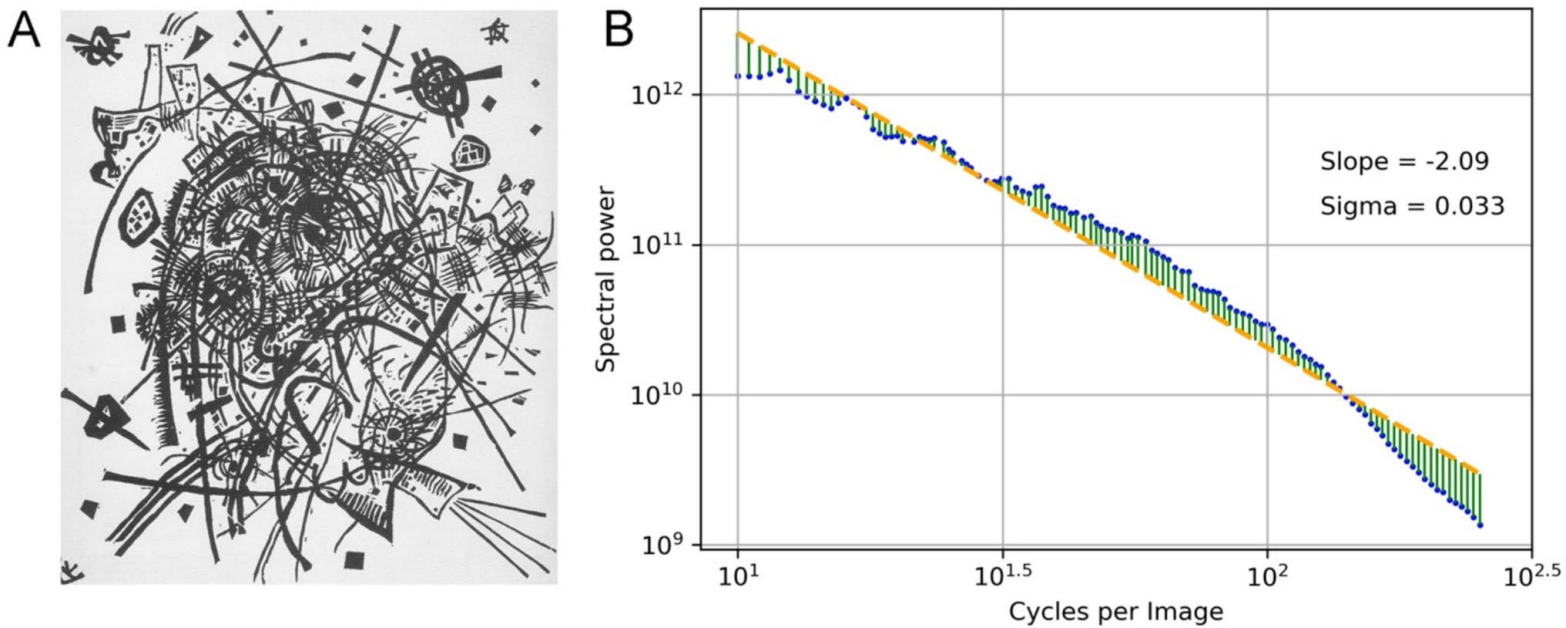
Calculation of Fourier spectrum sigma. *Notes*. **A** Woodcut by Vasily Kandinsky (Kleine Welten VIII, 1922; in the public domain). **B** Log-log plot of the Fourier power spectrum (*blue dots*) for the range of 10 to 256 cycles per image. The *yellow dashed line* is the fitted line. Fourier sigma is the sum of the squares of the deviations from the fitted line (*vertical green lines*), sampled at nearly regular intervals and divided by the number of data points (*blue dots*)

Another measure that can be derived from the log-log plot of Fourier power versus spatial frequency is the deviation of the data points from the regression line (Fig. 9). This measure, which has been called Fourier spectrum Sigma (Redies et al., 2020), is calculated as the sum of the squares of the deviations, divided by the number of data points. In the original version of the method (Redies et al., 2020; Redies et al., 2007) and in the present work, the deviations were summed for the original data points (no binning at equal intervals in the log-log space), which can lead to an overrepresentation of high frequencies. To reduce this overrepresentation, the Toolbox also offers another version that uses data points fitted at about equal intervals in the log-log plots (Fig. 9B), which corresponds to the method for fitting the regression line itself (Table 2). Results from the two versions correlate strongly for the 1000 images of the AVA dataset of artworks (Murray et al., 2012) (Pearson's coefficient of correlation *r* = 0.91).

The Sigma value indicates to what extent the Fourier spectrum deviates from a power law relationship. Higher values of Fourier spectrum Sigma correspond to larger deviations. Sigma values are generally low for natural images and large sets of traditional artworks, i.e., a straight line fits the log-log plots well (Graham & Field, 2007; Koch et al., 2010; Mather, 2014; Redies et al., 2007). Images perceived as unpleasant typically show deviations of the spectral curve from a straight line (Fernandez & Wilkins, 2008; O'Hare & Hibbard, 2011).

#### Fractal dimension (2D and 3D)

The concept of scale invariance in natural scenes and patterns can also be quantified using a geometric scaling parameter called the fractal dimension (*D*). Specifically, while the Fourier spectrum slope depends on the amplitude of intensity variations at different spatial scales (a photometric property), the fractal dimension depends on the density of such intensity variations (a geometric property). By examining the boundary edges between filled and empty regions within an image, *D* quantifies how detailed a pattern is at different spatial scales. *D* is thus an index of the relationship between the coarse and fine geometrical structure in a pattern. Patterns characterized by a low *D* value contain more coarse-scale structure, which yields a remarkably sleek and minimalist form. Conversely, fractals with a high *D* value correspond to patterns with predominantly fine spatial-scale structure, showcasing intricate and elaborate structures and detail (Taylor & Sprott, 2008).

It is important to emphasize that *D* is not a diagnostic tool to detect fractal patterns in the first place, but it is an image property that can be used to quantify the complexity of a pattern. In support of this notion, it has been found that the perceived complexity of a wide range of images (from natural scenes to paintings and synthetic patterns) all increase linearly with increasing *D* values (Bies et al., 2016a; Forsythe et al., 2011; Mureika & Taylor, 2013; Spehar et al., 2003; Viengkham & Spehar, 2018). Interestingly, *D* is inversely related to the Fourier spectrum Slope value, another measure that reflects the complexity of an image (see section "Fourier spectrum Slope"). A steeper Fourier spectrum slope is equivalent to a lower *D* value, and vice versa. A detailed comparison of the two techniques can be found elsewhere (Bies et al., 2016a; Fairbanks & Taylor, 2010; Spehar & Taylor, 2013).

One of the most frequently used techniques to determine the fractal dimension *D* of a pattern or an object is the box-counting method. The Toolbox contains two different variants of this method, the 2D algorithm used by Branka Spehar and colleagues (Viengkham & Spehar, 2018) and the 3D algorithm used by George Mather (Liu et al., 2014; Mather, 2018). Both techniques involve covering the pattern or the object with a grid of boxes/cubes of a certain size and counting how many of these boxes/cubes contain part of the pattern or the object. To estimate *D*, this process is repeated with different box/cube sizes to analyze the relationship between the box/cube size and the number of boxes/cubes needed to cover the pattern.

##### 2D *D* algorithm

**Fig. 10:**
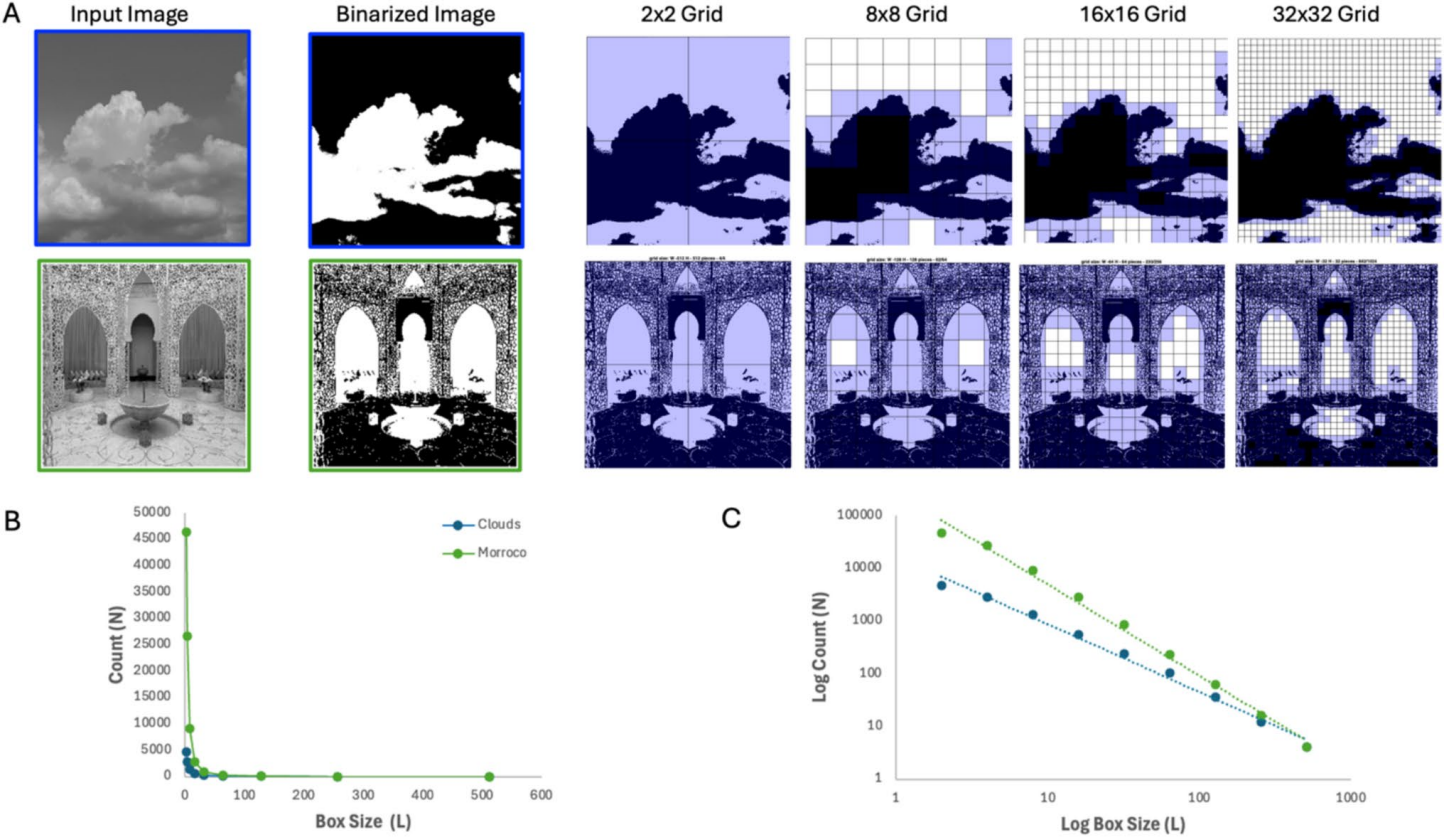
Illustration of the box-counting technique for calculating the 2D fractal dimension *D* for two different images. *Notes.* The input image (**A**) is binarized and then overlaid with a grid of varying, increasingly smaller, box sizes (*L*). The *purple* boxes in each grid correspond to the count of how many boxes in each grid contain binarized edges. **B** As *L* decreases, the number of boxes (*N*) required to measure the length of a boundary edge increases, following a power-law relationship. **C** When plotted in log-log coordinates, the slope of the resulting line corresponds to the 2D fractal dimension *D*

As emphasized earlier, the fractal dimension is independent of photometric properties of images, such as perceived brightness. Instead, it is based on the estimation of the density of spatial variations at different spatial scales. This is the reason why images are first binarized with respect to their mean lightness (or pixel intensity) before a box-counting procedure is applied, as illustrated in Fig. 10A. The binary image is then overlaid with a grid of varying, increasingly smaller box sizes (side length, *L*) within the grid to count how many boxes of the grid contain binarized edges within them (count, *N*). As illustrated in Fig. 10A, reducing the box size (i.e., using smaller values of *L*) is analogous to examining the image at finer (higher) spatial frequencies. The relationship between the box size (*L*) and the box count (*N*) follows a power law, as illustrated in Fig. 10B. In this context, the exponent *D* represents the fractal dimension, which can be quantified by plotting *log N* as a function of *log (1/L)* and fitting a linear regression (Fig. 10C). The slope of this regression line corresponds to the 2D *D*. Possible values for 2D *D* range between 1 (low complexity) and 2 (high complexity). On average, viewers prefer intermediate *D* values for a wide variety of natural and synthetic images (Taylor, 2006).

Edge-only image variations of algorithms to calculate 2D *D* can be used as well (Redies et al., 2015), and are based on edge extraction from the thresholded black-and-white images (Fig. 10A), e.g., by using the Canny algorithm (Canny, 1986). However, despite undergoing thresholding and edge extraction, the inherent fractal-scaling characteristics of images are preserved. This consistency renders fractal dimension *D* particularly appropriate as a measure of scale invariance across various image categories (Spehar & Taylor, 2013; Spehar et al., 2016; Viengkham & Spehar, 2018), including artworks.

##### 3D* D* algorithm

To measure the 3D fractal dimension *D* (Mather, 2018), image pre-processing consists of cropping each image to the largest central square region, which is then transformed into the L*a*b* (CIELAB) color space. From the Lightness channel of this space, the fractal dimension is calculated using an improved 3D box-counting algorithm (Jin et al., 1995; Li et al., 2009; Liu et al., 2014), which keeps the size of the image constant while changing the size of the boxes. This is unlike the 2D algorithm that keeps the size of the boxes constant as the image is resized to different magnifications (see above). The count of each individual box is the difference between the highest and lowest pixel lightness value. The 3D version of calculating *D* is based on a log-log plot of box size versus the sum of the counts of each box. The logarithm used here has base E. As for the 2D version, 3D *D* corresponds to the slope of the regression line in this plot. The 3D *D* value has a range from 2 (low complexity) to 3 (high complexity).

Note that 2D and 3D *D* versions correlate well (Figs. 1 and 3), with a Spearman’s coefficient *ρ* = 1.00 for the 1000 synthetic random-phase images (Branka Spehar, unpublished data), 0.62 for the JenAesthetic dataset of 1629 Western paintings (Amirshahi et al., 2015), and 0.68 for the 1000 random images from the AVA dataset of artworks (Murray et al., 2012).

### Measures of self-similarity inspired by vision models (PHOG and CNN)

Alternative measures of self-similarity have been developed that can be related more directly to models of visual system function. In the Toolbox, we include two such measures. One is derived from the Pyramid Histogram of Oriented Gradients (PHOG) descriptor (Fig. 2; Amirshahi et al., 2012; Bosch et al., 2007) and the other uses low-level features from a CNN (Fig. 4).

#### PHOG-based self-similarity

The PHOG method was originally developed for image classification and to describe spatial shape (Dalal & Triggs, 2005). A step-by-step explanation of the method can be found in the Appendix in to the publication by Braun et al. (2013).

To start with, the image size should be normalized for the PHOG analysis, e.g., to a total of 100,000 pixels (image width × image height; Braun et al., 2013) because the calculated Self-similarity values depend on image size (see Fig. 7 in Redies and Groß, 2013). Resizing can be carried out with a separate function of the Toolbox or in the popup menu of the PHOG method.

To calculate Self-similarity, images are first converted into the L*a*b* color space. The three channels are then combined in a gradient image (Fig. 2C), as described in the section "Complexity (HOG method)". Subsequently, a PHOG descriptor is generated for each image based on histograms of oriented gradients (HOG features; Fig. 2E). HOG features are obtained for equally sized orientation bins. Typically, 16 bins that cover 360 degrees are used (for example, see Fig. 2E). Initially, the HOG features are obtained for the entire image at the ground level (Level 0). Then, HOG features are calculated for consecutive levels of an image pyramid (PHOG; Bosch et al., 2007) up to a given level (e.g., up to Level 3; Fig. 2B, E). The pyramid is obtained by dividing the ground image into four rectangles of equal size (2 × 2 grid on Level 1; labeled I-IV in Fig. 2B). Each section at Level 1 is then divided again into four rectangles of equal size to obtain the next level of the pyramid, and so on (Fig. 2B). Accordingly, Level 2 contains 16 sections (4 × 4 grid) and Level 3 contains 64 sections (8 × 8 grid). HOG features have been generated up to Level 3 in most previous studies, which is also the top level for the PHOG analysis in the Toolbox. At higher levels, the calculated values become unstable, because the image sections used in the analysis are exceedingly small and the HOG features become noisy (Amirshahi et al., 2012).

The HOG features at Levels 1, 2, and 3, respectively, are compared to the HOG feature at the ground level (Fig. 2E) with the Histogram Intersection Kernel, which serves to calculate the degree of how similar two sets of HOG features are (range between 0 to 1). In the popup menu of the Toolbox, it can be specified which levels are included in the calculation and with what weight. For example, Self-similarity can be expressed as the mean value for Levels 1–3 of the pyramid, with equal weight for all levels (Redies & Brachmann, 2017; Redies et al., 2020).

Self-similarity is higher (closer to 1) if the HOG features at different levels of the pyramid are more similar to the ground-level HOG feature. Low values that approach 0 indicate minimal Self-similarity between the HOG features. Traditional paintings and other visually pleasing stimuli have an intermediate to high degree of Self-similarity (Braun et al., 2013).

#### CNN-based self-similarity

**Fig. 11:**
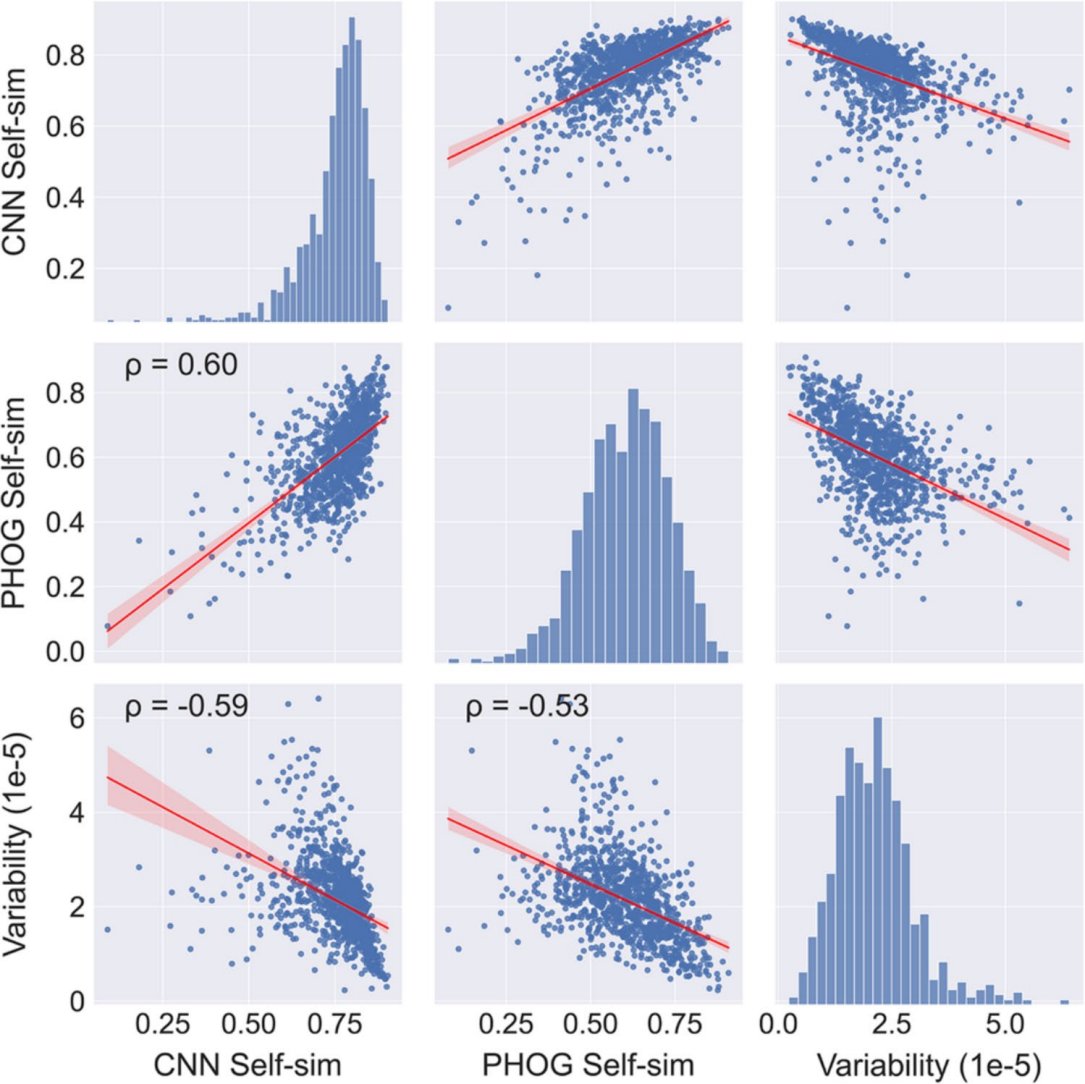
Scatter plots for the image properties that relate to self-similarity. *Notes.* Results for 1000 images randomly selected from the AVA dataset are shown. All correlations are significant (*p* < 0.05). The *red line* represents the result from linear regression with the 95% confidence interval indicated by red shading. Frequency histograms for each image property are shown along the diagonal axis. CNN Self-sim, CNN-based Self-similarity; PHOG Self-sim, PHOG-based Self-similarity; ρ, Spearman’s coefficient of correlation

In the section "CNN feature-based Symmetry", we introduced early convolutional layers of CNNs as models of human visual function (Fig. 4). We argued that, to model symmetry detection by humans, low-level CNN features are better suited than pixel-based methods that focus on specific image properties, such as luminance gradients (see section "Complexity (HOG method)”. A similar case can be made for Self-similarity. Brachmann and Redies (2017b) developed a CNN-based method to measure Self-similarity by simultaneously considering information about luminance edges, color, and spatial frequencies. The authors showed that the CNN-based method yielded responses that resemble human visual function more closely than responses obtained with the PHOG-based method. To measure Self-similarity, the network processes the image to generate the filter responses for the first convolutional layer (*conv1*) of an AlexNet pretrained on an ImageNet (Krizhevsky et al., 2012). From these results, histograms of maximum responses are produced over a grid of equally sized subsections of an image. Similar to the PHOG-based method, this histogram is then compared to the histograms of 64 subsections (8 × 8 grid, Level 3 in Fig. 2). Self-similarity is expressed as the median of all calculated values. A value closer to 1 indicates higher Self-similarity and a value closer to 0 lower Self-similarity. The scatter plots in Fig. 11 indicate that CNN-based Self-similarity correlates moderately with PHOG-based Self-similarity (ρ = 0.6).

### Feature distribution and entropy

As the last group of image properties, we will present algorithms that focus on the distribution of image features, such as pixel values, oriented gradients or edges, across an image. We will conclude with two image properties that are derived from low-level CNN features.

#### Homogeneity

**Fig. 12:**
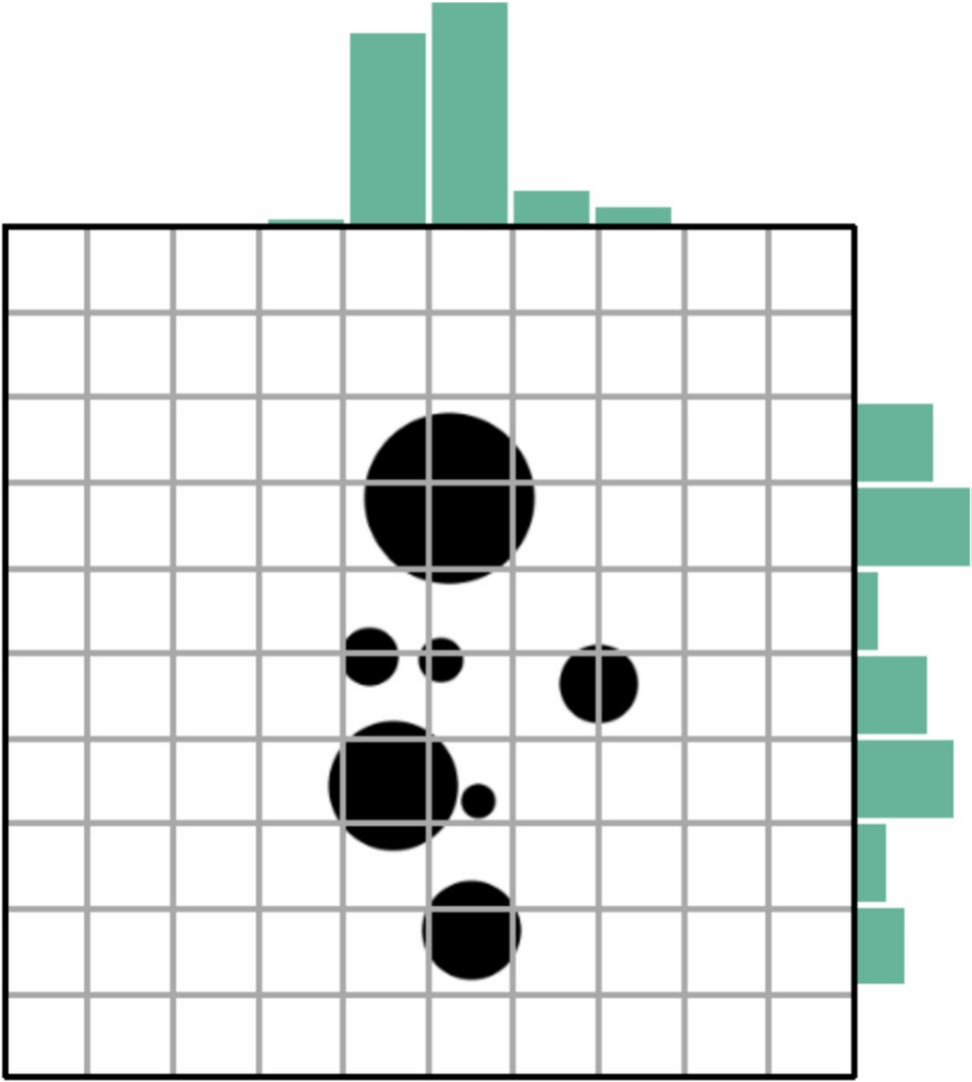
An example for the calculation of Homogeneity. *Notes.* A sample image from the stimulus set of the study by Hübner and Fillinger (2016) is overlaid with a 10 × 10 grid. The sums of the black pixels in the columns and rows are represented by a histogram above the image and to the right of the image, respectively (for details, see text)

Homogeneity is an image property that correlates moderately with Lightness entropy (Hübner & Fillinger, 2016). As introduced in the section “Lightness entropy”, the entropy of lightness (pixel intensity) values indicates how uniform the frequencies of lightness values are in an image. Lightness entropy is based on frequency histograms across all possible lightness values in an image. In contrast, to compute Homogeneity, the input image is first converted into a binary (black-and-white) image using the Otsu method (Otsu, 1979). The binary image is then divided into *n* × *m* subregions of equal size (in the Toolbox *n* = *m* = 10). An example of such a division is shown in Fig. 12. For each subregion, the number of black pixels is determined. The resulting *n* × *m* matrix contains the number of black pixels per subregion. With these numbers, the Shannon entropy is calculated by computing −*p*∙log_2_*p* for each subregion, where *p* is the corresponding proportion of black pixels. The values obtained are then summed across all subregions and the result is divided by the maximum entropy, which is defined by log_2_ of the total number of subregions. For the example in Fig. 12, the maximum entropy is log_2_(100) = 6.64. By multiplying the result by 100, one obtains the entropy as percentage. For the example, it is 59.1. As a percentage, Homogeneity can take values from 0 to 100. An image with exactly the same quantity of black pixels in each of its subregions would have a maximum Homogeneity value, while an image with black pixels in one subregion only and none in the others would have a minimum Homogeneity value.

Analogous to computing the Homogeneity across all subregions, Homogeneity can also be computed across rows and columns separately. For this objective, the number of black pixels in each row and column of the matrix are summed, resulting in a histogram for the vertical sums and the horizontal sums, respectively. The two histograms for the example are shown in Fig. 12. As can be seen, the distribution of black pixels is more even across the rows than across the columns. Computing the corresponding Homogeneities reveals values of 80.0 and 49.4, respectively. The final Homogeneity value that is returned by the current version of the Toolbox, is the average of the horizontal and vertical values. For the example in Fig. 12, this value is 64.7. Thus, unlike Lightness entropy, Homogeneity also captures the spatial distribution of pixels. For an image that has all black pixels in a subregion, Lightness entropy can still be relatively high, but Homogeneity is not. In their study of perceptual balance in geometrical images, Hübner and Fillinger (2016) showed that the homogeneity measure correlated substantially with ratings of balance and liking, as well as with the Balance measure and the DCM measure (for the AVA dataset, see Fig. 1).

#### Anisotropy of gradient orientation

Above, we described measures of Complexity and Self-similarity that can be derived from the Histograms of Oriented Gradients (HOG) descriptor (Dalal & Triggs, 2005). The same descriptor can also be used to determine how heterogeneously gradient strength is distributed across orientations in an image, a property here called Anisotropy (Braun et al., 2013).

To calculate this property, a gradient image is obtained, as described in section "Complexity (HOG method)”. From the gradient image, the Toolbox generates histograms of the oriented gradients (HOGs) for the 16 equal-sized orientation bins and the 64 subregions at Level 3 of the HOG pyramid (Fig. 2E). Anisotropy is calculated as the standard deviation between the summed and normalized gradient strengths for each orientation bin of all subregions (Braun et al., 2013). Low anisotropy means that gradient strength is uniform across orientations, i.e., all orientations tend to be of equal strength in the image. High anisotropy means that orientations differ in their overall prominence, e.g., for the photograph in Fig. 2A where vertical orientations are more prominent than the other orientations.

In general, traditional Western artworks tend to be less anisotropic than diverse types of non-art images (Melmer et al., 2013; Redies et al., 2012).

#### Edge-orientation entropy (EOE)

**Fig. 13:**
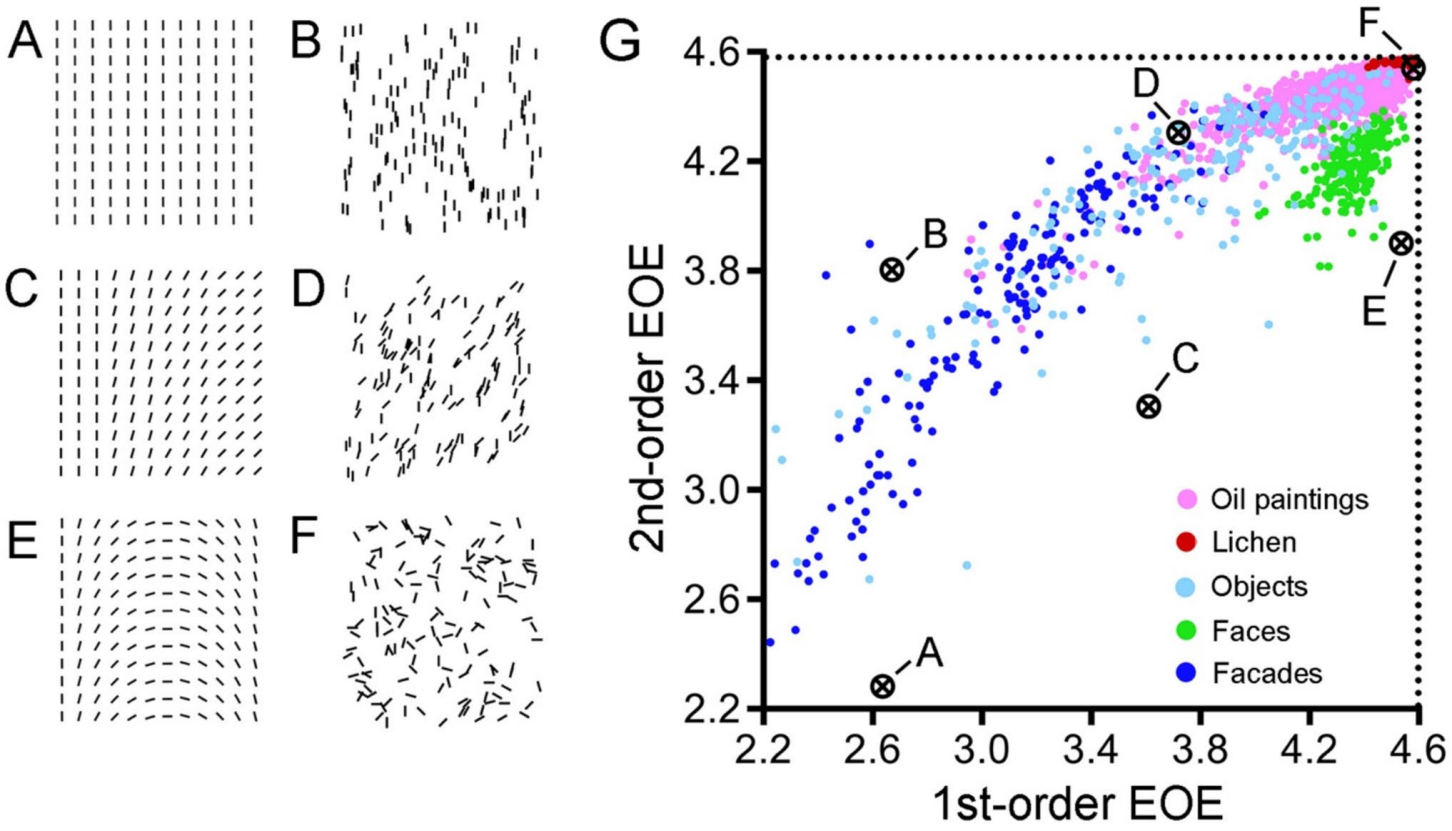
First- and second-order edge-orientation entropy (EOE) in simple line patterns (A–F) and different types of images (G). *Notes.* The EOE values of the line patterns shown in (**A**–**F**) are marked by the *black symbols* in the scatter plot (**G**). EOE values for different types of images are shown in different colors, as indicated by the *colored dots* on the right-hand side in (**G**). Adapted from Redies et al. (2017) with permission

Next, we describe another method to study the distribution of edge orientations in an image by measuring the Shannon entropy of edge orientations. This method is based on edge-filtered images and refers to the uncertainty of encountering a particular orientation in an image at a particular position (Geisler et al., 2001). Synthetic images to illustrate this method are shown in Fig. 13A–F.

To calculate edge-orientation entropy (EOE), the Toolbox extracts edges by applying a bank of 24 oriented Gabor filters, which covers one full rotation (360 degrees) when combined. Gabor filters are akin to simple cell responses in the primary visual cortex (Mehrotra et al., 1992). By applying oriented Gabor filters to an image, summary statistics of edge orientations can be obtained.

First-order EOE is defined as the Shannon entropy of the summary orientation histogram that represents the full spectrum of edge orientations for the entire image (Redies et al., 2017). First-order EOE measures how uniformly the edge orientations are distributed across the full spectrum of orientations in the entire image. First-order EOE is high if all orientations are present at about equal strength in the image. As an example, Fig. 13E, F shows synthetic patterns composed of short lines segments with an equal number of lines for all orientations. First-order EOE assumes intermediate values for a less homogenous histogram of edge orientations (Fig. 13C, D), and it is low if all lines have the same orientation (Fig. 13A, B; see plot in Fig. 13G). Note that, in all cases, first-order EOE is independent of the arrangement of the lines in the image (regular pattern in Fig. 13A, C, E and random pattern in Fig. 13B, D, F).

**Fig. 14:**
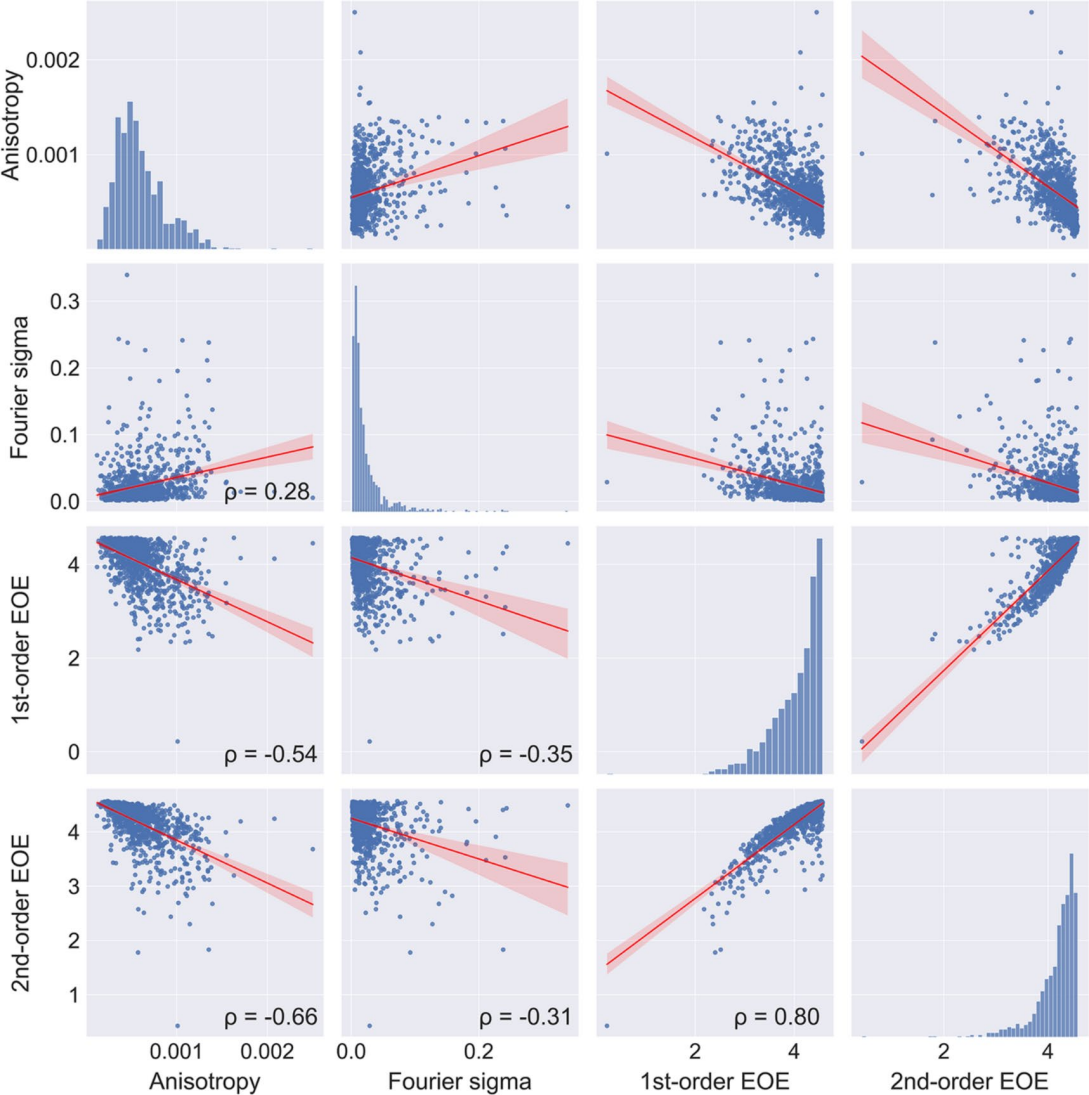
Scatter plots for the image properties that relate to feature distribution. *Notes.* Results for 1000 images randomly selected from the AVA dataset are shown. All correlations are significant (*p* < 0.05). The *red line* represents the result from linear regression with the 95% confidence interval indicated by *red shading*. Frequency histograms for each image property are shown along the diagonal axis. EOE, edge-orientation entropy; ρ, Spearman’s coefficient of correlation

Second-order EOE (Geisler et al., 2001; Redies et al., 2017) is a measure of how independent the spatial positions of edge orientations are across an image; unlike first-order EOE, it also captures the spatial arrangement of the edges in the image. To obtain second-order EOE, the orientation of each strong edge in an image is related pairwise to the orientation of all other strong edges in the image. To avoid local regularities, such as collinearity, edge pairs of less than 20 pixels distance are excluded from the analysis. Histograms of the pairwise orientation *differences* are then obtained for all (strong) edge pairs in an image. Second-order EOE is maximal if all orientation differences between line pairs occur at equal frequencies in the histograms (Fig. 13F). In that case, edge orientations are distributed independently of each other in the image, i.e., the edge orientation at one position in an image has no relation to the orientation at other position (Fig. 13B, D, F). Second-order EOE is lower if the histograms of edge orientation differences are more heterogeneous due to spatial regularities in the line pattern (Fig. 13A, C, E). Note that for second-order EOE to be high, first-order EOE must be high as well (Redies et al., 2017). First- and second-order EOE correlate strongly with each other (ρ = 0.8). They also correlate weakly to strongly with other measures that reflect deviations from a uniform distribution of oriented gradients or edges across orientations (Anisotropy and Fourier Sigma; Figs. 1 and 14; see also section "Fourier spectrum Slope").

Due to computational restrictions, we limit the number of edges for pairwise analysis to the 10,000 strongest edge responses for the calculation of the second-order EOE. Despite this limitation, second-order EOE is by far the computationally most expensive image property in the Toolbox.

The motivation for using these measures in aesthetics research was twofold. First, the perceptual analysis of the spatial layout of contours and edges plays an important role in contour grouping and object recognition in natural images (Geisler et al., 2001). For artworks, some artists and art theorists have argued that the spatial layout of pictorial elements (“composition”) is also an important determinant of visual appreciation (Arnheim, 1954; Locher et al., 1999; Redies et al., 2017). Indeed, large subsets of artworks of different cultural provenance display high EOE values (Geller et al., 2022; Redies et al., 2017), indicating that they have relatively homogenous orientation histograms (Fig. 13G). Other examples for images with high first-order EOE or second-order EOE are photographs of particular types of natural objects, such as lichen growth patterns (Fig. 13G), but also other man-made patterns, such as synthetic line patterns and decorated building facades (Grebenkina et al., 2018; Stanischewski et al., 2020).

Second, the distribution of edges across orientation histograms carries information about how angular or curved stimuli are (see section “Fourier spectrum Slope”). In general, curved stimuli have more homogenous edge-orientation histograms, giving rise to higher EOE values, than angular stimuli (Grebenkina et al., 2018; Stanischewski et al., 2020; Watier, 2024). It is well established that curved stimuli are also liked more than angular ones (Bar & Neta, 2006; Bertamini et al., 2016; Chuquichambi et al., 2022). Relatively high EOE values may thus be a hallmark not only of traditional artworks, but also of other types of visually pleasing stimuli (Grebenkina et al., 2018).

#### CNN feature variances

The image properties that we described above reflect particular aspects of image structure, without accounting for overarching similarities and interactions in their response statistics, such as variations in their frequency and spatial distribution. To carry out a more comprehensive and combined analysis of different types of visual response features, Brachmann and colleagues (2017) proposed to analyze variances of CNN filter responses at the first convolutional layer (*conv1*) of a CNN (AlexNet; Krizhevsky et al., 2012). As mentioned above (see section on “CNN feature-based Symmetry”), features from the *conv1* layer of AlexNet resemble neural responses in the early mammalian visual cortex in that they process rather diverse types of information, such as oriented edges, color-opponent blobs, and spatial frequency information (Fig. 4). In particular, about half of the AlexNet feature maps display color-opponent characteristics. Low-level CNN features can thus encompass color features in a more comprehensive way than many of the other image properties (e.g., the Fourier slope or the [P-]HOG-based measures).

Here, we describe two derivatives of a CNN, i.e., two types of variance of *conv1* features in the AlexNet (Brachmann et al., 2017). For the calculation of the two variances, each feature map is partitioned into *n* × *n* subregions of equal size. Responses are then recorded in each subregion for each filter by a max-pooling operation over the response maps.

First, we calculate the total variance for each *conv1* feature over all 96 filter entries of the* n* × *n* subregions of *conv1*. This variance represents the mean variance of all filter responses in all subregions of an image. Intuitively speaking, this variance has been interpreted as *sparseness* in a wider sense; it has been interpreted as the inverse of *richness* of filter responses across an image and assumes lower values if more filters respond with similar strength in more subregions of an image (Brachmann et al., 2017).

Second, we calculate the median over the variances of each of the 96 filters, again for *n* × *n* subregions at *conv1*. This variance is high if there is a high degree of *variability* of filter responses across the response maps, whereas lower values suggest less variability of filter responses or higher *self-similarity* (Brachmann et al., 2017). Intuitively speaking, this variance is higher if feature responses are more diverse in the subregions of an image. As would be expected, Variability correlates inversely with CNN-based Self-similarity and PHOG-based Self-similarity (Fig. 11).

**Fig. 15:**
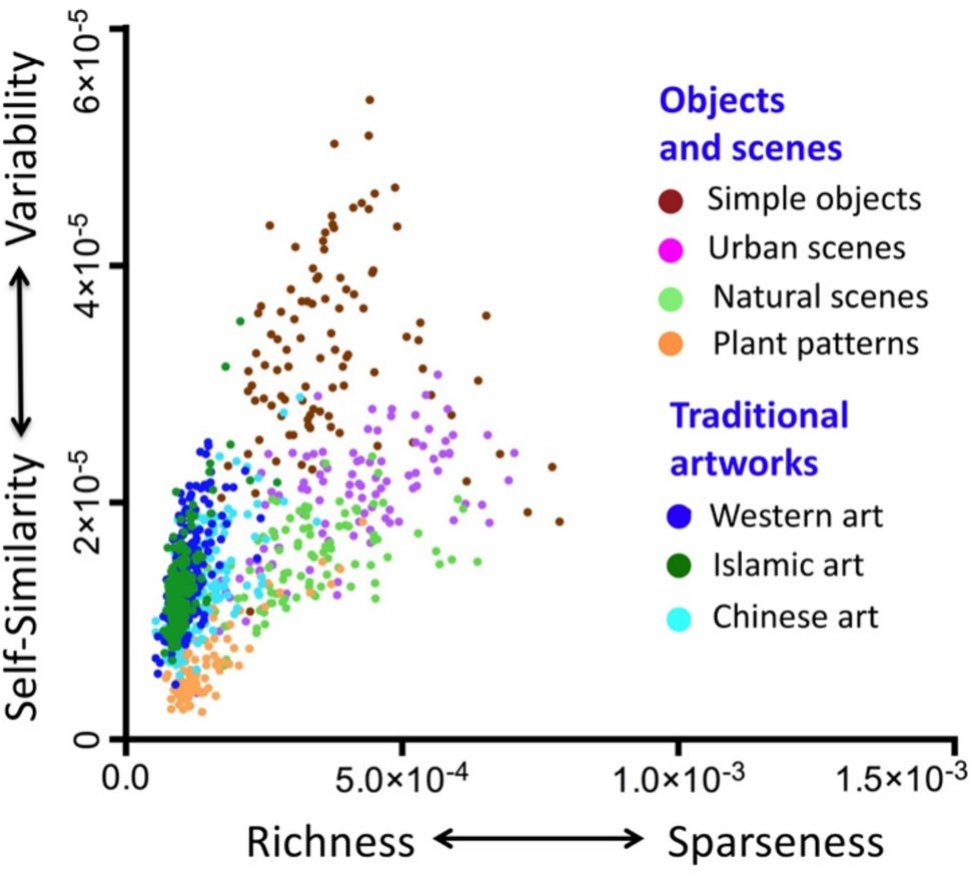
CNN-derived variances of feature responses to photographs of objects and scenes (non-art images) and to images of artworks. *Notes.* Each *dot* represents the variances calculated for one image of a traditional artwork from different cultural provenance (Western, Islamic, and Chinese) or non-art stimuli (photographs of simple objects, urban scenes, natural scenes, and plant patterns; as indicated by the different colors). The two variances were calculated for the 96 filters of the first convolutional layer (conv1) of an AlexNet network (Krizhevsky et al., 2012; see Fig. 4). Adapted from Brachmann et al. (2017) with permission

The two variances differ for diverse types of natural and man-made images (Brachmann et al., 2017). Large subsets of traditional artworks of diverse cultural provenance (Western, Islamic, and Chinese) are characterized by a high Richness (or a low Sparseness) of filter responses and an intermediate to high degree of Variability (or an intermediate to low degree of Self-similarity; Fig. 15). Other types of images, e.g., photographs of simple objects, urban scenes, natural scenes and plant patterns, exhibit different ranges for the two variances, and there is little overlap with the traditional artworks (Fig. 15).

### Technical considerations

#### Translation of original scripts to Python 3

Original scripts to compute image properties with the Toolbox were gathered from various researchers, including Ronald Hübner (University of Konstanz), George Mather (University of Sussex), Branka Spehar (University of New South Wales), and Christoph Redies and Ralf Bartho (University of Jena). Many of these scripts were originally written in Python 2 or MATLAB. Python 2 has not been maintained since January 1, 2020; the current Python version is 3.12 as of March 2024. Python 2 is no longer supported by most modern Python packages. MATLAB is a proprietary software that requires a paid license. Furthermore, the built-in functions of MATLAB are not open source and the exact structure of its algorithms is thus not transparent to the user. Therefore, we translated all of the scripts to Python 3.

Care was taken to ensure that the Python 3 versions produced the same results as the original Python 2 and MATLAB scripts. A near-perfect match was accomplished for all image properties, except for HOG Complexity and Anisotropy. For these two properties, the original MATLAB implementation allowed users to automatically scale the images prior to calculation. It was not possible to transfer this resizing operation one-to-one to Python 3 because the MATLAB *resize* function does not return the same results as similar Python implementations (e.g., Pillow, scikit-image, or opencv, see also Watier (2024)). Furthermore, MATLAB source code is also not easily reverse-engineered into Python because it is not open source. Without initial resizing, however, the Toolbox version of these image properties gives the same results as the original MATLAB version. Therefore, resizing is still possible in the Toolbox version, but the results are different from those of the original MATLAB versions.

In most of the original versions of the scripts, the documentation provided in the code was rather limited. In the new Python 3 versions, extensive documentation is available for each individual image property. Nevertheless, the structure and form of the novel scripts (names of functions, classes, etc.) still reflect the original implementations. The GitHub website contains all Python 3 scripts for calculating the image properties and the graphical user interfaces that were built with streamlit. It also comprises the original MATLAB and Python 2 scripts.

In the following sections, we will point out several technical issues that more generally pertain to calculating image properties and to running the Toolbox in an efficient way.

### Running the Toolbox efficiently

#### System requirements

The Toolbox runs natively in the browser on all end devices. There are two ways in which the Toolbox can be used: First, a cloud version is available at https://aesthetics-toolbox.streamlit.app/ and allows the toolbox to be used without local installation but with limited resources, i.e., the memory and computing power are comparatively low here. This is why the cloud version is primarily suitable for calculating image properties for small data sets and for testing the toolbox. With the cloud version, the images have to be uploaded to the Streamlit Community Cloud and calculated there, which can be problematic in terms of image copyrights. Second, the Toolbox can be installed locally. The maximum number of images that can be calculated simultaneously and the computing time depend on the resources of the local system. With the local installation, the browser is used as an interface only and no data is uploaded to the Internet.

#### Multithreading

Ideally, in view of the increasing amount of data to be analyzed, it would be desirable for the Toolbox to support multithreading on modern multi-core CPUs. Unfortunately, the common operating systems (Mac OS, Linux, and Windows) handle multithreading differently, so that the Toolbox's requirements for performance collide with its goal of platform independence, which was prioritized for the design of the Toolbox. For large data sets (larger than 6 GB), the user should install the local Toolbox version and divide the images to be calculated into several sets and start a separate local instance of the application in the browser for each set. In the background, the respective operating system will automatically assign the instances of the Toolbox that run in parallel to the logical CPU threads. The GitHub site also contains a script-only (no GUI) version of the Toolbox that can be used for local multi-threading or deployment to an HPC cluster.

#### Computational load: Dependence on image size and image property

Two important factors in the performance of the Toolbox are the size of the images and the image properties selected. The number of pixels in an image increases with the square of its side length. In general, the computation time for most image properties in the Toolbox scales linearly with image resolution for simpler metrics, such as image size. For more complex properties, such as edge-orientation entropy, the time requirement grows polynomially. In addition, hardware constraints, such as CPU cache size, can contribute to disproportionately longer computation times as image resolution increases.

A good heuristic approach is to scale the input images to the resolution that has also been used to collect the aesthetic ratings for the images (if applicable). Note that even a high-resolution image will automatically be downscaled to the maximum resolution of the display when displayed. This kind of scaling would lead to sufficiently small images (common display size of 1920 × 1080 pixels) for which the image properties can be calculated in a reasonable amount of time. Also note that resizing has a strong effect on many image properties, as described above. The same image may yield very different values for a given image property at different resolutions.

In addition to calculating image properties, the toolbox provides a graphical interface for many common image pre-processing methods. These include: (a) image cropping, (b) image padding, (c) image color rotation, and (d) image resizing options. For example, the methods can be used to resize all images to a similar image size by resizing the longer side of rectangular images to 512 or 1024 pixels and adjusting the shorter side accordingly to maintain the aspect ratio. Note that some image properties require a fixed image size for their computation (e.g., [P-]HOG-based Complexity or Edge-orientation entropy; see the online documentation for these image properties). For example, the image resolution is set to a fixed size of 512 × 512 pixels for the input image of CNN-based algorithms. Therefore, when calculating these image properties, such peculiarities should be taken into account for the choice of image pre-processing.

Furthermore, the individual image properties have very different computational demands. Image properties such as the simple mean values or standard deviations of the color channels are relatively fast to compute. The measures based on CNN features and the PHOG measures (Complexity, Anisotropy, and Self-similarity) are computationally more intensive. By far the most demanding measure is the second-order EOE, which compares the orientation of each edge in the image with all other edges (for the 10,000 strongest edges).

The revised Python 3 scripts of the Toolbox are partially optimized for runtime. For EOE, there is a separate additional C++ implementation in Cython. This version can easily achieve a speedup factor of > 100 on modern machines (https://github.com/RBartho/C-version-2nd-Order-Edge-Orientation-Entropy).

## Discussion

The study of quantitative (objective) image properties and their role in aesthetic evaluations by human observers has been one of the central research topics in the field of experimental and computational aesthetics during the last 20 years or so. For the Aesthetics Toolbox, we selected 43 of these image properties and make algorithms to calculate them accessible to a broader audience. One of our aims is to enable researchers with no or little programming experience to calculate the image properties. To facilitate its usage, the entire Toolbox is written in one programming language (Python 3) and is built as a web application with the streamlit package (https://streamlit.io).

A major benefit of the newly created Toolbox is its simple-to-use interface, which runs platform-independent on most devices. The interface is intuitive and allows users to select images from the local hard drive for calculation, set the desired image properties and parameters, and export a single CSV file with the complete results. The Toolbox can be used as a cloud version, but local installations are also possible. In particular, for local installation, the browser is used only as an interface and the application runs only on the local computer. No data is uploaded to the Internet and no external server is involved. This feature helps to avoid the uploading of large images, which could put a heavy load on the available bandwidth, and to get around copyright issues that could possibly arise if protected images are uploaded to external servers.

The Aesthetics Toolbox includes image properties that cover widely different aspects of vision. Nonetheless, it still provides only a fraction of the image properties that have been studied in aesthetics research to date. Also, there are many different concepts for capturing aspects of vision that are rather divergent even if they carry the same name. For example, the methods for measuring symmetry in the Toolbox mirror rather different aspects of images and should be compared with caution (Fig. 5). By the same token, Madan et al. (2018) found that coefficients of correlations between different complexity measures range from 0.60 to 0.82 (see also Fig. 3). Such values are relatively low and raise the question of whether studies that use different complexity measures really analyze the same visual phenomenon (Madan et al., 2018; Van Geert & Wagemans, 2020, 2021). Moreover, calculated (objective) complexity does not always reflect the subjective impression of complexity of beholders (Forsythe et al., 2011; Marin & Leder, 2013; Nath et al., 2024; Zhou et al., 2023), which can be influenced by other subjective factors, such as familiarity with the stimuli or affect (Madan et al., 2018; Marin & Leder, 2013; McCormack & Gambardella, 2022). Zhou et al. (2023) demonstrated that ratings of subjective complexity relate more strongly to higher-level brain activity than to activity in early visual cortex; liking ratings correlate with higher-level (but not low-level) brain activity.

The way in which the different measures are implemented adds another layer of complexity. During the development of the Toolbox, it became evident that simple implementation details can have a large impact on the calculated values of the individual image properties. Such details include image pre-processing (resizing and cropping), file formats (effect of different RGB color formats or non-lossless image compression), differences in the custom functions of the programming languages used, and even variations in the different versions of a single Python package used. From a scientific point of view, this situation is unfortunate because it makes it difficult to compare research results from different groups. To reach an agreement on common measures (and their consistent implementation) across the diverse research communities seems out of reach in the near future. Possible reasons for this situation are the lack of open access to used scripts, difficulties in using scripts, lack of programming knowledge, costly software licenses for proprietary code, or lack of maintenance of the code.

The Aesthetics Toolbox is our contribution to improve this situation. On the one hand, it provides the research community with a user-friendly tool to calculate a large selection of image properties. On the other hand, the Toolbox is designed as an open-source project. Other scientists are hereby invited to add their concepts and scripts to the Toolbox and to develop it further, be it by adding more image properties, or by integrating additional functionality. To facilitate such contributions, the source code for the entire Toolbox is available under MIT licenses on GitHub (https://github.com/RBartho/Aesthetics-Toolbox). The GitHub project page also contains detailed installation instructions for the most popular operating systems (Windows, Mac, and Linux).

The Aesthetics Toolbox will eventually be accompanied by a searchable list of relevant datasets for aesthetics research (Lisa Koßmann, Ralf Bartho, Christoph Redies, and Johan Wagemans, unpublished data). This list will contain comprehensive information about each dataset and will allow researchers to find suitable datasets for their research projects using simple search criteria.

## Data Availability

Data and supplementary materials for the present work are available at github.com/RBartho/Aesthetics-Toolbox.

## Footnotes

1 https://github.com/RBartho/Aesthetics-Toolbox

## Code availability

The complete code for the toolbox is available at github.com/RBartho/Aesthetics-Toolbox.
